# Telomere-to-telomere genome assembly of *Phaeodactylum tricornutum*

**DOI:** 10.7717/peerj.13607

**Published:** 2022-07-05

**Authors:** Daniel J. Giguere, Alexander T. Bahcheli, Samuel S. Slattery, Rushali R. Patel, Tyler S. Browne, Martin Flatley, Bogumil J. Karas, David R. Edgell, Gregory B. Gloor

**Affiliations:** 1 Department of Biochemistry, Schulich School of Medicine and Dentistry, Western University, London, Ontario, Canada; 2 Suncor Energy, Sarnia, Ontario, Canada

**Keywords:** *Phaeodactylum tricornutum*, Genome assembly, Nanopore sequencing, Telomere-to-telomere, High-molecular weight DNA, Methylation, Transposons

## Abstract

* Phaeodactylum tricornutum* is a marine diatom with a growing genetic toolbox available and is being used in many synthetic biology applications. While most of the genome has been assembled, the currently available genome assembly is not a completed telomere-to-telomere assembly. Here, we used Oxford Nanopore long reads to build a telomere-to-telomere genome for *Phaeodactylum tricornutum*. We developed a graph-based approach to extract all unique telomeres, and used this information to manually correct assembly errors. In total, we found 25 nuclear chromosomes that comprise all previously assembled fragments, in addition to the chloroplast and mitochondrial genomes. We found that chromosome 19 has filtered long-read coverage and a quality estimate that suggests significantly less haplotype sequence variation than the other chromosomes. This work improves upon the previous genome assembly and provides new opportunities for genetic engineering of this species, including creating designer synthetic chromosomes.

## Introduction

*Phaeodactylum tricornutum* is a marine diatom that is described as a “diatom cell factory” (Butler, Kapoore & Vaidyanathan, 2020) because it can be used to manufacture valuable commercial products. Recent genetic toolbox expansions, such as delivering episomes by bacterial conjugation (Karas et al., 2015), CRISPR-editing tools (Russo et al., 2018b; Moosburner et al., 2020; Sharma et al., 2018; Stukenberg et al., 2018; Slattery et al., 2018; Serif et al., 2018), the generation of auxotrophic strains (Zaslavskaia et al., 2000; Buck et al., 2018; Slattery et al., 2020), and the identification of highly active endogenous promoters (Erdene-Ochir et al., 2019) are enabling rapid implementation of new product designs into commercial-scale production.

The genome of *P. tricornutum* CCAP 1055/1 was sequenced in 2008, and resulted in a scaffold-level assembly predicting 33 chromosomes (NCBI assembly ASM15095v2) (Bowler et al., 2008). Chloroplast and mitochondrial genomes have also been published (Oudot-Le Secq et al., 2007; Oudot-Le Secq & Green, 2011), and have previously been identified as targets for genetic engineering (Cochrane et al., 2020), as well as other chromosomes (Karas et al., 2013). Although the Bowler et al. assembly contains several telomere-to-telomere chromosomes, many scaffolds have only zero or one telomere, suggesting they are either incomplete or fragments of another chromosome. More recent work identifying centromeric sequences (Diner et al., 2017) in *P. tricornutum* has suggested that there may be less than 33 chromosomes.

While the current assembly is an excellent resource, it does not represent a completed genome assembly. The lack of a completed genome assembly for *P. tricornutum* means that synthetic biology researchers are unable to pursue generating artificial chromosomes with this model diatom. The full sequence of each chromosome is required to rebuild chromosomes by DNA synthesis. It is also important to know the location and sequence of mobile genetic elements that could be removed to in order to simplify a potential fully synthesized chromosome sequence. A more complete understanding of the genome will be a resource to help researchers answer more fundamental biological questions about *P. tricornutum*.

To generate a telomere-to-telomere assembly of *P. tricornutum* CCAP 1055/1, we used a hybrid approach with ultra-long reads from the Oxford Nanopore MinION platform and highly accurate short reads from the Illumina NextSeq platform. We also introduce a novel graph-based approach to manually resolve telomere-related assembly errors. This approach identifies all unique telomere sequences and we demonstrate how it can be applied to manually correct assembly errors adjacent to chromosome ends. The full structural context of the *P. tricornutum* genome provides additional information for potential synthetic biology applications to manipulate the genome of this diatom cell factory.

## Methods

### Growth

*Phaeodactylum tricornutum* (Culture Collection of Algae and Protozoa CCAP 1055/1) was grown in L1 medium without silica at 18 °C under cool white fluorescent lights (75 mE m^−2^ s^−1^) and a photoperiod of 16 h light:8 h dark as described previously (Slattery et al., 2018).

### DNA extraction

200 mL of culture (approximately 5 ×10^8^ cells) was spun at 3000×g for 10 min at 4 °C. The pellet was resuspended in one mL TE (pH 8.0) and added dropwise to a mortar (pre-cooled at −80 °C) pre-filled with liquid nitrogen. The frozen droplets were ground into a fine powder with a mortar and pestle, being careful to keep the cells from thawing by adding more liquid nitrogen as necessary. The frozen powder was transferred to a 15 mL Falcon tube where two mL of lysis buffer was added (1.4 M NaCl, 200 mM Tris–HCl pH 8.0, 50 mM EDTA, 2% (w/v) CTAB, RNAse A (250 µg/mL) and proteinase K (100 µg/mL)). The solution was mixed very slowly by inversion, incubated for 30 min at 37 °C (mixed very slowly halfway through incubation). Cellular debris was pelleted at 6000×g for 5 min. Lysate was transferred to a new 15 mL Falcon tube. One volume of 25:24:1 phenol:chloroform:isoamyl alcohol was added, mixing slowly by inversion. The sample was centrifuged at 6000×g for 5 min. The aqueous phase was transferred as slow as possible to a new Falcon tube. One volume of 24:1 chloroform:isoamyl alcohol was added, and mixed slowly with end-over-end inversion. The sample was centrifuged at 6000×g for 5 min. Approximately 450 uL of the aqueous phase was transferred into new 1.5 mL Eppendorf tubes. To the Eppendorf tube, 1/10 volume of 3 M NaAc pH 5.2 and two volumes (final volume) of ice-cold 100% ethanol were added, mixing slowly by end-over-end inversion. The sample was centrifuged at 16,000×g for 5 min, and washed twice with 500 uL 70% ethanol. Ethanol was decanted, and the pellet was dried for approximately 10 min by inverting on a paper towel. The pellet was resuspended in 100 uL 10 mM Tris–HCl pH 8.0, 0.1 mM EDTA pH 8.0. After resuspending overnight at 4 °C, DNA fragments less than 20 kbp were then selectively removed using the Short Read Eliminator (SRE) kit from Circulomics (Baltimore). DNA from the same extraction was used for sequencing on both the Oxford Nanopore MinION and Illumina NextSeq 550 platform.

### Sequencing

An Oxford Nanopore MinION flow cell R9.4.1 was used with the SQK-LSK109 Kit according to the manufacturer’s protocol version GDE_9063_v109_revK_14Aug2019, with one alteration: for DNA repair and end-prep, the reaction mixture was incubated for 15 min at 20 °C and 15 min at 65 °C. Basecalling was performed after the run with Guppy (Version 3.6). NanoPlot (De Coster et al., 2018) was used to generate Q-score *versus* length plots and summary statistics. The read N50 of the unfiltered reads was approximately 35 kb (Supplemental Figure S1).

For Illumina sequencing, the Nextera XT kit was used, and a 2X75 paired-end mid-output NextSeq 550 library was prepared according to the manufacturer’s protocol, and sequenced at the London Regional Genomics Center (lrgc.ca). Reads were trimmed using Trimmomatic v0.36 (Bolger, Lohse & Usadel, 2014) in paired-end mode with the following settings: AVGQUAL:30 CROP:75 SLIDINGWINDOW:4:25 MINLEN:50 TRAILING:15. SLIDINGWINDOW AND TRAILING were added to remove poor quality base calls.

### Assembly

#### Telomere identification

We first obtained sequences for the end of every linear chromosome. The sequence of the telomere repeats for *P. tricornutum* are known from the previous assembly (Bowler et al., 2008) to be repeats of AACCCT. All long reads larger than 50 kilobases with three or more consecutive telomeric repeats (or the reverse complement) were extracted by filtering using NanoFilt (De Coster et al., 2018) and by string matching using grep. All-versus-all mapping of the telomeric reads was performed using minimap2 (Li, 2016). Only overlapping reads with a minimum query coverage of 95% were retained.

To determine the sequence of unique telomeres for each chromosome, a network graph was generated with iGraph (Csardi & Nepusz, 2006). Each read name was used as a vertex, and edges were generated between each overlapping read with more than 95% query coverage. Noise was filtered by removing any group of overlaps with less than 5×coverage. There were 93 vertices that had greater than 20×coverage; that is, there are 93 unique telomere sequence groups. Most groups had approximately 40×coverage (number of long reads per group), however, several outliers had more than 60×coverage. These represent duplicated regions in the telomeres that are not unique (*i.e.,* more than one haplotype or chromosome contains this sequence). The longest read of each telomere group, typically greater than 100 kb in length, was retained as a representative telomere sequence for correction. Example code for this is available in Supplemental Code S1.

#### Assembly

Miniasm was chosen to reduce computational power needed over other assemblers like Canu (Koren et al., 2017) or Flye (Kolmogorov et al., 2019). Nanopore reads longer than 75 kilobases were used for initial assembly with miniasm, (Li, 2016) using the parameters -s 30,000 -m 10,000 -c 5 -d 100,000. From this initial assembly, the output from miniasm were manually completed with the following approach:

(1) Mapping of telomeric reads against the unitig. If no telomere was present on the unitig and a high query coverage alignment was found, the unitig was extended to the telomere sequence of the mapped telomere. (2) After telomere extension (or confirmation), reads longer than 50 kb were mapped to the unitig to confirm overlapping coverage over the entire chromosome. Coverage was evaluated using only reads larger than 50 kb and with higher than 50% query coverage, with an alignment score:length ratio less than two (similar to previous validation methods) (Giguere et al., 2020). A query coverage of only 50% was chosen to allow for potential haplotype divergence. (3) Telomere-to-telomere unitigs with overlapping ultra-long read coverage and no gaps were deemed validated and brought forward to improve base accuracy by read polishing.

The chloroplast and mitochondrial genomes were assembled using a reference based approach by first extracting all reads that aligned to the reference chloroplast and mitochondria with high query coverage. Reads were then *de-novo* assembled using miniasm.

#### Polishing

Due the repetitive nature of the genome and the diploid nature of *P. tricornutum*, raw assemblies were polished using an iterative approach with racon (Vaser et al., 2017), medaka (Oxford Nanopore) and Pilon (Walker et al., 2014) as described in the Methods section. Briefly, after each polishing iteration, we corrected errors that were introduced by the polishing algorithms as described in Supplemental File S1, and modified the medaka polishing by filtering reads using a minimum of 50% query coverage. The assembly was first polished by nanopore reads only, followed by Illumina read polishing using Pilon. For the chloroplast and mitochondria, the subset of reads identified as either chloroplast or mitochondria were used for polishing. The genome assembly is available on GenBank under accession GCA_914521175.

### Methylation

A total of 5mC methylation sites were predicted using Megalodon v2.2.1 (Oxford Nanopore Technologies) using the model res_dna_r941_min_modbases_5mC_CpG_v001.cfg from the Rerio repository (Oxford Nanopore Technologies) with Guppy 4.5.2. A default threshold of 0.75 was used as a minimum score for modified base aggregation (probability of modified/canonical base) to produce the final aggregated output. The percentage of reads methylated at the predicted locations are plotted in Supplemental Figure S2.

## Results

### Workflow

**Figure 1:**
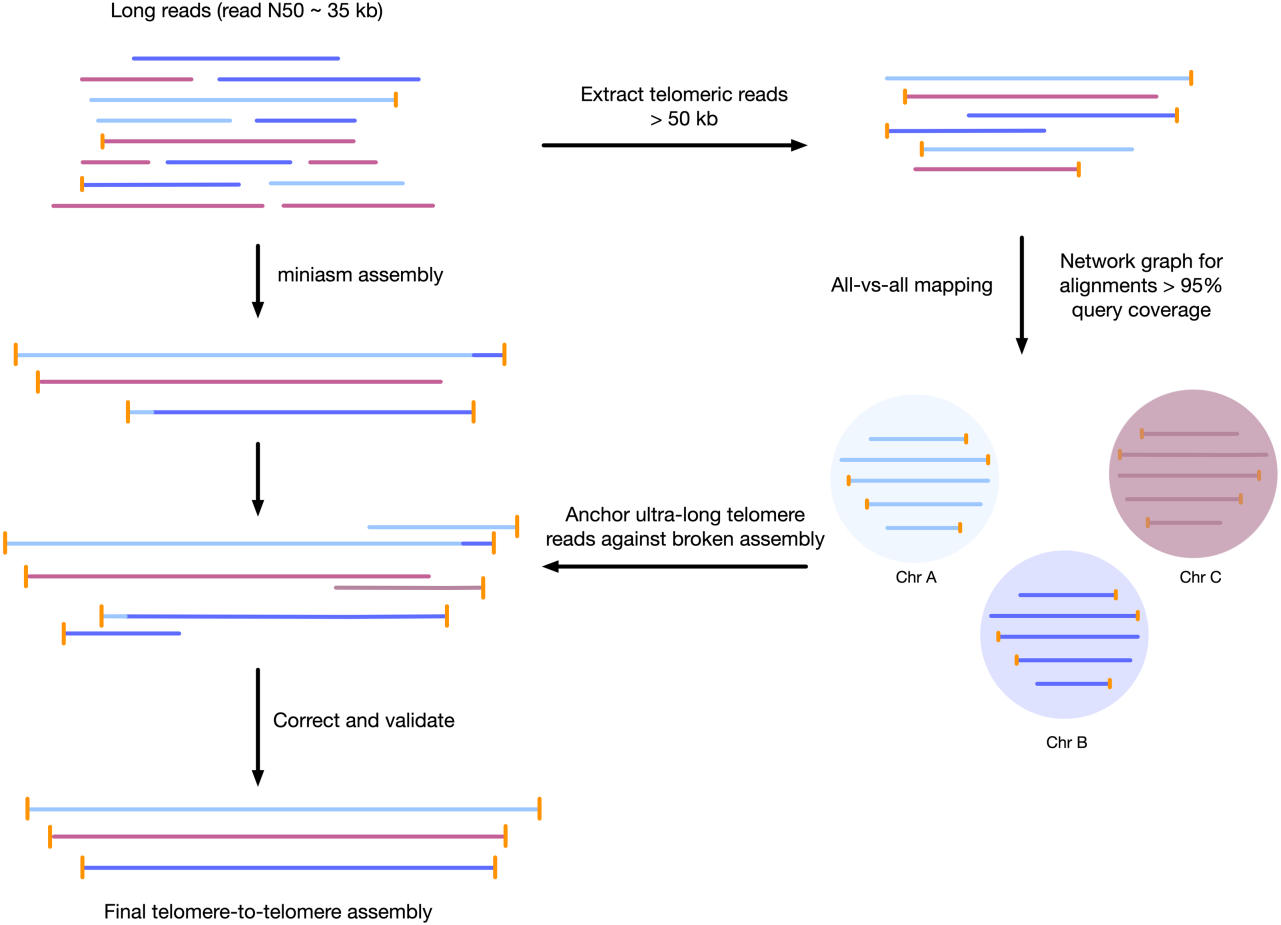
Workflow for telomere-to-telomere genome assembly. Telomere-containing nanopore reads larger than 50 kb are extracted and mapped in all-vs-all mode using minimap2. The resulting alignments are filtered by 95% query coverage, and a network graph is created using iGraph using read names as vertices, and alignments between reads as edges. Each resulting cluster represents one end of a chromosome. On a chromosome-by-chromosome basis, ultra-long read coverage is plotted. If an assembled chromosome is missing a telomere or has an assembly error revealed by a lack of overlapping read coverage, the longest read from each telomere cluster is mapped against the chromosome, and the resulting telomere is used to manually correct the assembly and extend to the telomere using an overlap-layout consensus approach.

We developed a sample preparation protocol that provided high-molecular weight DNA. We observed a read N50 of 35 kilobases, with the longest reads just over 300 kb, following sequencing with the Oxford Nanopore MinION platform. Of the 7.8 gigabases of raw sequence data, approximately 2.5 gigabases were from reads longer than 50 kilobases (Supplemental Figure S1). We found that chromosomes assembled using standard approaches were often mis-assembled around telomeres, or were fragmented and only contained 1 telomere. To correct each contig, we used the unique ultra-long telomere reads as described in the Supplemental File S1 and in Fig. 1. This approach was used to manually identify a tiling path for each chromosome until each chromosome was contiguous from telomere to telomere, and validated by a tiling overlapping read path.

#### Tiling path of overlapping reads verify contiguity

To ensure our genome assembly is contiguous, we generated multiple independent minimum tiling paths of overlapping long reads (Data S4, Fig. S2). Reads larger than 50 kb were mapped against the assembly using minimap2. To ensure no incorrect alignments were retained, any reads with less than 90% of the read aligned to the assembly were removed. From this subset, five independent minimum tiling paths that required at least 10 kb of overlap between each read were generated. All chromosomes have multiple independent (*i.e.,* no common reads) tiling paths of reads with a minimum overlap of 10 kb in the final assembly (five independent paths shown in PAF format (Li, 2016) format in Supplemental File S2), indicating that all chromosomes are contiguous. Chromosomes were manually corrected to meet this standard if necessary.

In addition to overlapping reads, Supplemental Figure S2 also shows the GC content for each chromosome. A previous study has proposed that centromeres could be identified by low GC content calculated in 100 bp windows (Diner et al., 2017). The 100 base window(s) with the minimum GC content are shown in Supplemental File S2, highlighted in red. These windows represent putative centromere sequences as previously described (Diner et al., 2017).

#### Telomere-to-telomere assembly comprises previous scaffolds

We ultimately obtained 25 telomere-to-telomere chromosome assemblies that recruit 98% of long reads, and these chromosomes comprise all previously proposed chromosomes from Bowler et al. (2008), as well as circularized chloroplast and mitochondrial genomes. The median coverage for unfiltered long reads across the nuclear genome was 202X, while median coverage for the chloroplast and mitochondrion were approximately 6201X and 528X, respectively. This was calculated in 1000 base windows using mosdepth (Pedersen & Quinlan, 2018).

**Figure 2:**
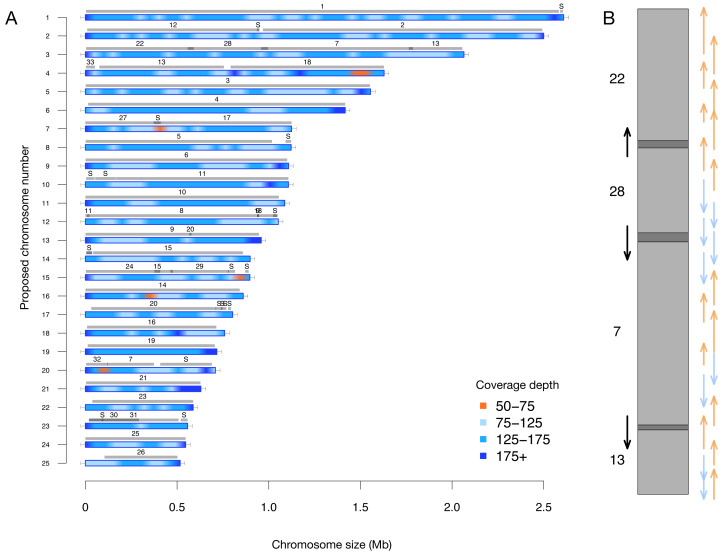
(A) Filtered long-read coverage and comparison to previous assembly. Reads longer than 20 kb were mapped against the assembly, filtered (minimum 20,000 base alignment and 50% query coverage), and genome coverage was calculated in 50 kb windows using mosdepth. The colours and ranges bottom-right) describe the coverage depth calculate for each 50 kb window. Newly proposed chromosomes names are indicated on the left (by length). Scaffolds from the previous genome assembly (ASM15095v2) are overlayed as grey bars, aligned using minimap2 in asm5 mode and filtered to retain minimum 10 kb alignments. Numbers on top of gray bars indicate which previous scaffold number, with S representing small “bottom drawer” scaffolds. Horizontal “T” bars on each end indicate telomere-repeat presence. (B) Visualization of proposed chromosome 3 with alignments to previous chromosomes. Dark gray regions indicate overlap. Coloured arrows on the right indicate minimum overlapping read path (orange = negative strand, blue = positive strand), black arrows on left show ultra-long reads that completely span regions where previous assembly could not assemble through.

A key feature of this updated assembly is the consistency with previous sequencing efforts (Bowler et al., 2008). Previously, 25 centromere sequences were identified (Diner et al., 2017), suggesting that there were fewer than the proposed 33 chromosomes. This agrees with our conclusion of 25 nuclear chromosomes. We independently resolved the location of all the previously proposed partial chromosomes without internal inconsistencies in Fig. 2 (*i.e.,* scaffolds with only one telomere were resolved into a telomere-to-telomere chromosome).

### Estimating the number of chromosomes using ultra-long reads

Previous studies have suggested that *P. tricornutum* has a minimum of 33 chromosomes using pulsed-field gel electrophoresis (Filloramo et al., 2021). Our orthogonal, reference-free method using network graphs of telomere-containing overlapping ultra-long reads revealed 25 chromosomes.

We used two properties of telomeres for this: first, telomeres on linear chromosomes can be identified by unique subtelomeric sequences, and second, that telomere-containing DNA fragments will begin or end with a telomere, representing the start or end of a chromosome. After aligning all telomere-containing reads and retaining only alignments with greater than 95% query coverage, we used iGraph to create network graphs, which resulted in two classes of independent graphs. The first class had 85 independent graphs, each with approximately 50 nodes (*i.e.,* 50 ultra-long reads in each graph), and the second class had eight graphs with approximately 100 nodes (Supplemental Figure S3). In a diploid organism we expect four telomeres per chromosome if we assume that each chromosome has two haplotypes; *i.e.,* (maternal + paternal) ×haplotypes. Under this assumption, 85 independent graphs with approximately 50 nodes represents 21.25 telomeres. Some chromosomes will not have diverged sufficiently, meaning there will be only two telomeres with twice the sequencing coverage per chromosome (maternal + paternal). The remaining eight graphs with 100 nodes each therefore gives a further four chromosomes.

With this logic we estimate 25.25 chromosomes exist in *P. tricornutum*, which agrees very closely with our final assembly of 25 chromosomes. The additional 0.25 chromosome may be explained by mitotic recombination (Bulankova et al., 2021). Using the features of ultra-long reads at the ends of linear DNA elements (*i.e.,* eukaryotic chromosomes) thus enables an orthogonal method for estimating the number of chromosomes in a reference-free manner.

### Assembly quality

To assess the quality of the assembly, we used Merqury (Rhie et al., 2020) to estimate the base-level accuracy and completeness by k-mer frequency, shown in Supplemental File S3. We found that the estimated quality value (estimated log-scaled probability of error for the consensus base calls by Merqury) ranged from 27–53, depending on the chromosome. The mean quality value (QV) for nuclear chromosomes was 28.86, with chromosome 19 as an outlier at 43. The QV for all nuclear genomes except for 19 are likely lower because the chromosomes were polished using heterozygous reads. The chloroplast and mitochondrial genomes have a QV of 53 and 42, respectively. Importantly, the k-mer completeness estimate of 80% suggests that many k-mers in the Illumina reads are not represented in this genome assembly, implying significant haplotype variation. This was also the case when using the Bowler assembly as input for Merqury.

We also estimated the genome completion using BUSCO (Manni et al., 2021). Using the stramenopiles_odb10 model, we found our assembly was 95% complete, with only 3% of expected BUSCOs missing. When evaluating the chromosome scaffolds of the Bowler assembly, we found it was 96% complete with 3% of expected BUSCOs missing.

After removing Lambda spike-in reads with NanoLyse, we found that 98.12% of long reads are recruited by the assembly. When reads are filtered by removing any read that does not align over more than 90% of it’s length (*i.e.,* query coverage is higher than 90%), the number of reads recruited drops to 74%.

### Filtered long-read coverage for Chromosome 19 is inconsistent with diploid state

We observed that chromosome 19 has remarkably consistent (*i.e.,* no drops in coverage) filtered long-read coverage relative to the other chromosomes (Fig. 2, Supplemental Figure S2). While we initially predicted *P. tricornutum* would have two haplotypes since it is diploid, recent work has demonstrated that while each cell has two haplotypes, many haplotypes within a population arise due to mitotic recombination (Bulankova et al., 2021). The consistency of filtered long read coverage for chromosome 19 indicates that there is only a single haplotype, whereas the other chromosomes have two or more haplotypes present, which can be inferred from inconsistent read depth at regions where haplotype divergences occur in Fig. 2 and Supplemental Figure S2. This indicates that there are not two haplotypes for chromosome 19, suggesting a different recent history for this chromosome.

### 5mC methylation and transposable elements

**Figure 3:**
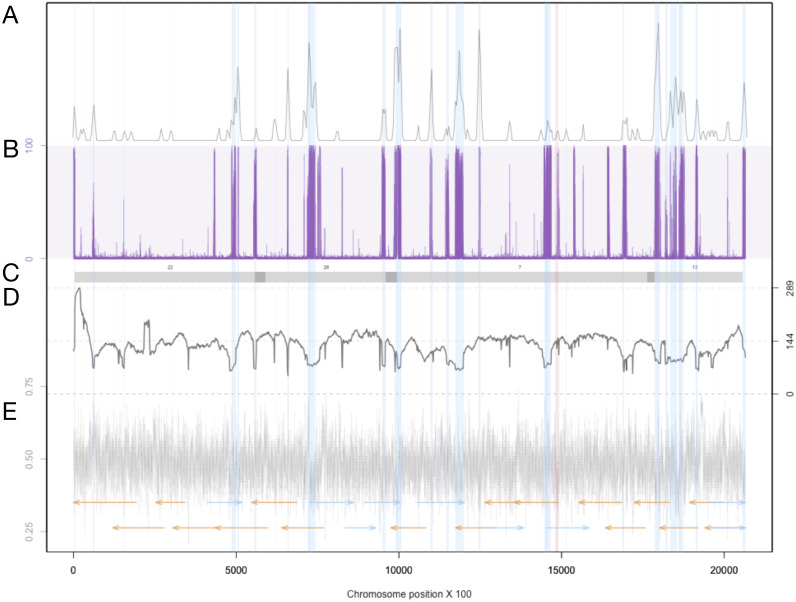
Summary of genomic features for chromosome 3. (A) The density of LTR-retrotransposons as predicted by the EDTA pipeline. (B) The proportion of reads that were called as methylated at each position along the chromosome. (C) Scaffolds from the previous assembly are overlayed in gray bars, with dark grey representing overlapping regions. (D) Filtered long-read coverage (minimum 20 kb length and 70% query coverage). (E) GC content calculated and plotted in 100 base windows. An overlapping read tiling path, with a minimum overlap of 30 kb, is shown with orange indicating reads mapping to the negative strand and blue indicating reads mapping to the positive strand. The region highlighted in red is the window with the lowest GC content.

The Extensive de-novo TE Annotator (EDTA) pipeline (Ou et al., 2019) was used to predict transposable elements in the genome. We found that the majority of transposable elements are long terminal repeat (LTR) retrotransposons (3.43% of the genome was found to be Copia-type, 5.86% were unknown, while terminal inverted repeats were only 1.17% of the genome, and helitrons were 0.54% of the genome). Each LTR region is represented as a shaded blue region in Supplemental Figure S2 in blue, and density plots of the end locations are shown in the top quadrant. Chromosome 19 contained the fewest transposable elements at 50. The locations and density of LTR-retrotransposons are plotted in Fig. 3 for proposed Chromosome 3 and Supplemental Figure S2 for all other chromosomes.

Previous studies have found that some tranposable elements were hypermethylated (Veluchamy et al., 2013). Using chromosome scale nanopore methylation basecalling, we found a strong signal between many predicted LTR retrotransposons and methylation status (Fig. 3, Supplemental Figure S2). To test this, we enumerated all chromosome positions with methylated sites and transposons, and performed a Fisher’s Exact Test, resulting in a *p*-value of 2.2e−16.

We examined the association between LTR transposon dense regions and regions where the previous assembly failed to generate overlapping regions. We observed that scaffolds with overlapping regions (Supplemental Figure S2) generally were not assembled into full chromosomes because of ambiguity in the placement of the LTR-rich regions at the ends of the scaffolds. These are now resolved by the long-read assembly identified here. Additionally, many of the low-coverage regions of our assembly overlap with the locations of the LTR-dense regions, consistent with chromosomal rearrangements being more likely in these regions. Further investigation at these regions is required.

## Discussion

Here, we developed a graph-based approach to locate the unique telomere ends of all *P. tricornutum* chromosomes, and applied this information to generate an telomere-to-telomere assembly. The new assembly incorporates all the previous chromosome fragments from Bowler et al. (2008).

The chromosomes show marked variations in sequencing coverage that can be explained by haplotype variation. Where haplotype variation occurs, filtered long reads will not align against the assembly. This suggests that there are large regions of the chromosomes that have substantial haplotype differences. Strikingly, only chromosome 19 has completely consistent coverage between the telomeres. While this needs to be further investigated, we speculate that this chromosome in this strain may have undergone a recent sequence homogenization event. Previous work has also found that the same chromosome appears homozygous in the wild type strain (Russo et al., 2018b; Bulankova et al., 2021). It has previously been speculated that *Phaeodactylum tricornutum* may be capable of sexual reproduction (Mao et al., 2020; Patil et al., 2015), but there has yet to be conclusive evidence of this occuring.

Chromosome 19 has a high quality value of 43, while the other nuclear chromosomes have lower quality values around 28. For all chromosomes except 19, this drop in per-base quality is due to polishing the nanopore assembly with a heterozygous read set. However, the high quality value and consistent filtered-long read coverage suggest that there are not highly divergent haplotypes for chromosome 19. Recently published data has demonstrated that mitotic recombination occurs frequently in *P. tricornutum* (Bulankova et al., 2021). They independently showed that there is a significantly lower SNP density on chromosome 19, agreeing with this finding, in addition to Russo et al. (2018b). Interestingly, the high rate of mitotic recombination suggests that it is unlikely that a static haplotype-resolved diploid genome may be fully resolved for this species with currently available technology. In this context, the k-mer completeness estimate we obtained from Merqury suggests that up to 20% of the Illumina k-mers result from SNPs arising from mitotic recombination events within the population, suggesting a high degree of haplotype divergence.

We demonstrate that nanopore sequencing can identify methylated regions, and the entire methylome of *P. tricornutum* is strongly associated with transposable elements (Supplemental Figure S2). This agrees with previous work (Veluchamy et al., 2013) that found a significant enrichment of DNA methylation at LTR retrotransposons, and we provide an updated map by predicting methylation sites directly from sequenced native DNA.

We have deposited all short and raw long-read data publicly for use by the community as Project PRJEB42700 on the European Nucleotide Archive. This telomere-to-telomere genome assembly will be a resource for designing and creating synthetic chromosomes in *Phaeodactylum tricornutum*, as well as answering fundamental biological questions for this species.

## Conclusions

Here, we report a collapsed telomere-to-telomere genome assembly for *Phaeodactylum tricornutum* CCAP 1055/1. A combination of ultra-long nanopore sequencing reads (greater than 100 kb), a novel approach to correcting assembly errors near telomeres, and manual curation enabled the completion of a telomere-to-telomere genome. We also describe a method to estimate the number of chromosomes using the properties of ultra-long telomere-containing reads in a reference-free manner. We provide the signal level nanopore data as a resource to enable the community to further investigate 5mC methylation for this species. This work improves our upon our current understanding of the model diatom *Phaeodactylum tricornutum* to enable further developments in synthetic biology.

## Supplemental Information

10.7717/peerj.13607/supp-1 Supplemental Information 1 Quality for nanopore sequencing experiment (A) Read length *vs.* average read quality. (B) Yield by length. (C) Run information. NanoPlot was used to produce figure and summary statistics.

10.7717/peerj.13607/supp-2 Supplemental Information 2 Summary for every chromsome For each chromosome, the density of LTR-retrotransposons as predicted by the EDTA pipeline are plotted in the top quadrant. The proportion of reads that were called as methylated at each position along the chromosome are plotted in purple. Scaffolds from the previous assembly are overlayed in gray bars, with dark grey representing overlapping regions. Filtered long-read coverage (minimum 20 kb length and 70% query coverage) was plotted as a black line. GC content was calculated and plotted in 100 base windows in the bottom quadrant. An overlapping read tiling path, with a minimum overlap of 30 kb, is shown with orange indicating reads mapping to the negative strand and blue indicating reads mapping to the positive strand. The regions that are annotated at LTR-retrotransposons are highlighted in light blue.

10.7717/peerj.13607/supp-3 Supplemental Information 3 Histogram of telomere graphs Telomere graphs were separated into two classes (1) graphs with between 20 and 75 reads, representing normal sequencing depth and (2) graphs with more than 75 reads, representing 2X normal sequencing depth.

10.7717/peerj.13607/supp-4 Supplemental Information 4 Quality value estimates for each chromosome provided by Merqury

10.7717/peerj.13607/supp-5 Supplemental Information 5 Five independent tiling paths using long reads were created with a minimum 10 kilobase overlap for each overlap

10.7717/peerj.13607/supp-6 Supplemental Information 6 Methods for manual curation of genom

10.7717/peerj.13607/supp-7 Supplemental Information 7 Example code for chromosome number estimates Pseudo-code to describe method for creating network graphs.

## Additional Information and Declarations

### DNA Deposition

The following information was supplied regarding the deposition of DNA sequences: All sequencing data are available on the European Nucleotide Archive under project PRJEB42700.

### Data Availability

The following information was supplied regarding data availability: The data files used to generate figures are available at GitHub: https://github.com/dgiguer/phaeodactylum-tricornutum-genome and at Zenodo: Daniel Giguere. (2021). dgiguer/phaeodactylum-tricornutum-genome: first release for submission (0.1). Zenodo. https://doi.org/10.5281/zenodo.4731049. The code to reproduce the telomere identification workflow is available in the Supplementary File.
