# Supplementary material for: Telomere-to-telomere genome assembly of Phaeodactylum tricornutum
Source: PeerJ. 2022 Jul 5;10:e13607. doi: 10.7717/peerj.13607 (PMC9266582; doi:10.7717/peerj.13607)
Supplement: Supplemental Information 1 — (A) Read length vs. average read quality. (B) Yield by length. (C) Run information. NanoPlot was used to produce figure and summary statistics. [file peerj-10-13607-s001.pdf]

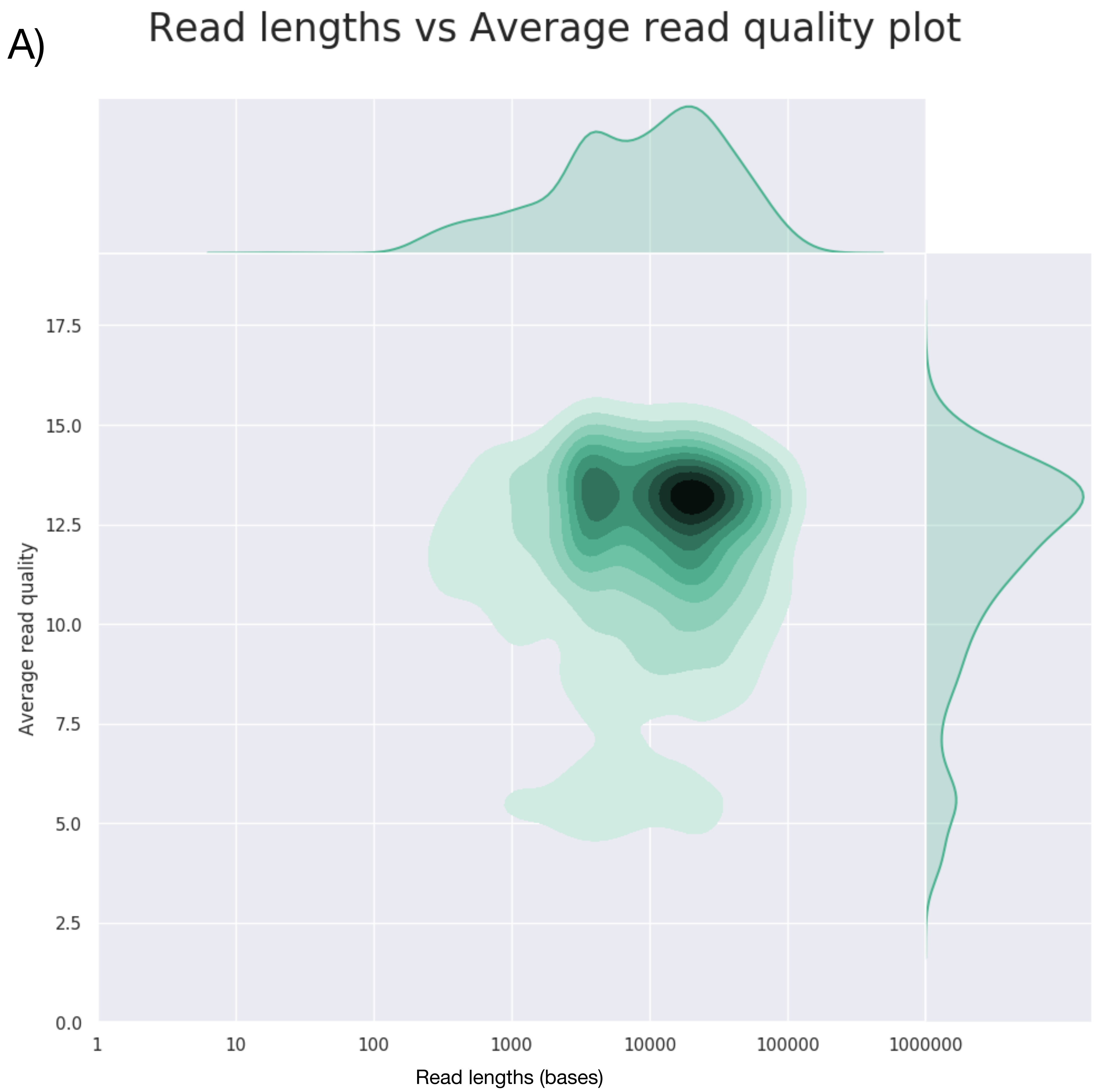

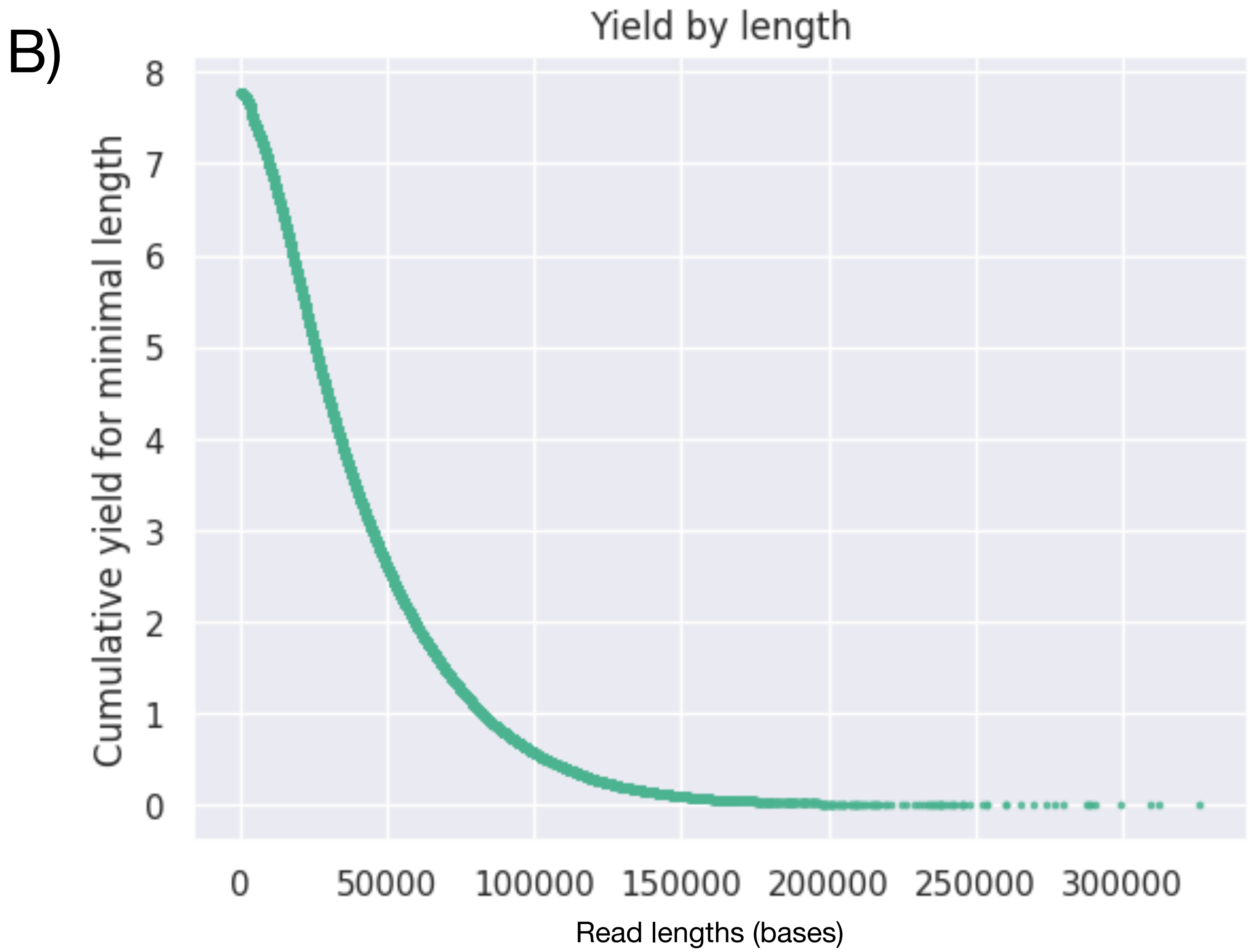

C)

Read length N50:	35,035
Number of reads:	438,929
Median read quality:	12.3
Total bases:	7,785,091,135

Supplemental Figure S1: Quality of Nanopore sequencing data. A) Read length vs. average read quality calculated by NanoPlot (axis titles are modified). B) Yield by length vs. read length as reported by NanoPlot (axis titles are modified). C) Run information as reported by NanoPlot.
