# Supplementary material for: Telomere-to-telomere genome assembly of Phaeodactylum tricornutum
Source: PeerJ. 2022 Jul 5;10:e13607. doi: 10.7717/peerj.13607 (PMC9266582; doi:10.7717/peerj.13607)

Chromosome 1

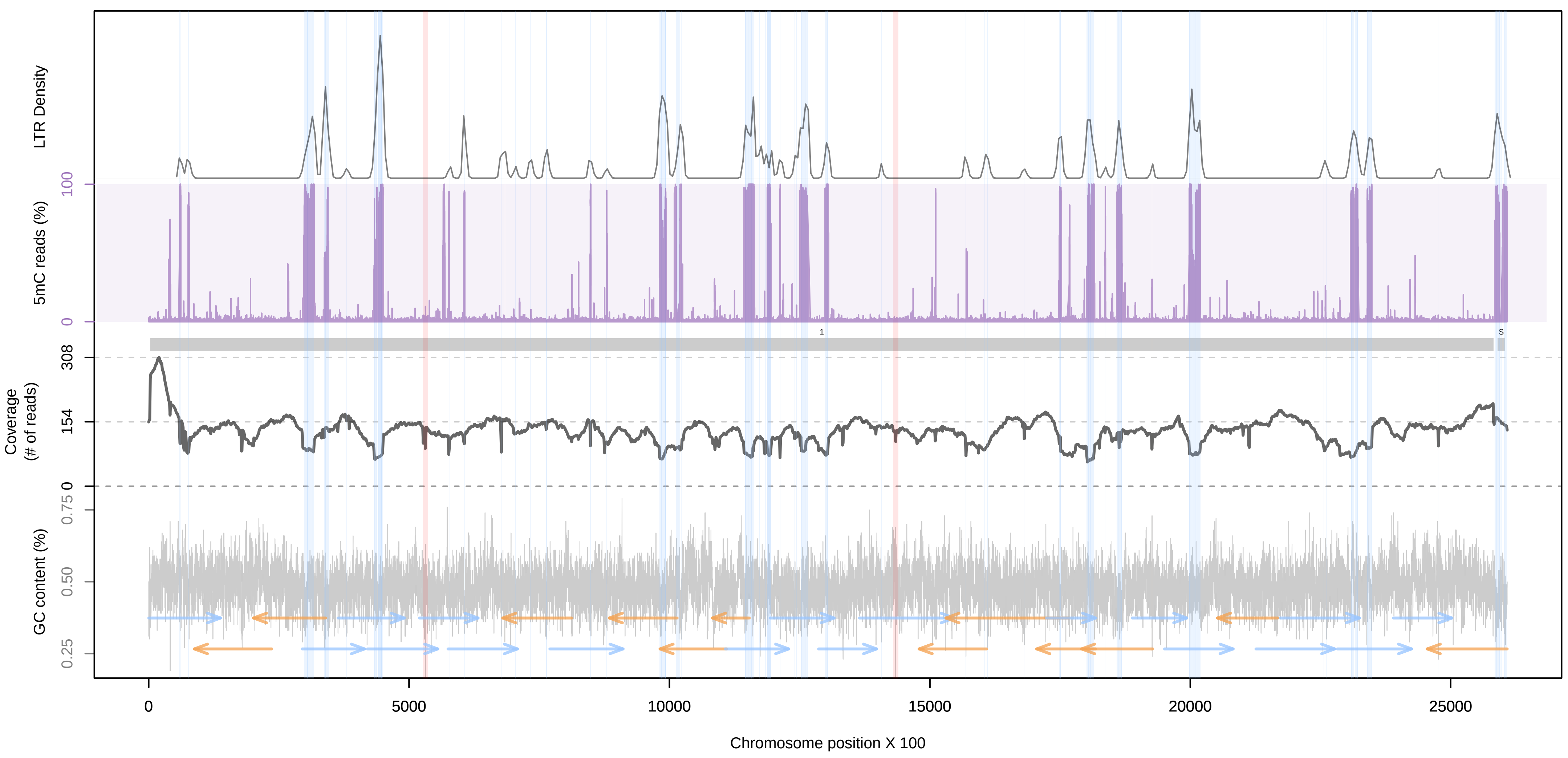

Chromosome 2

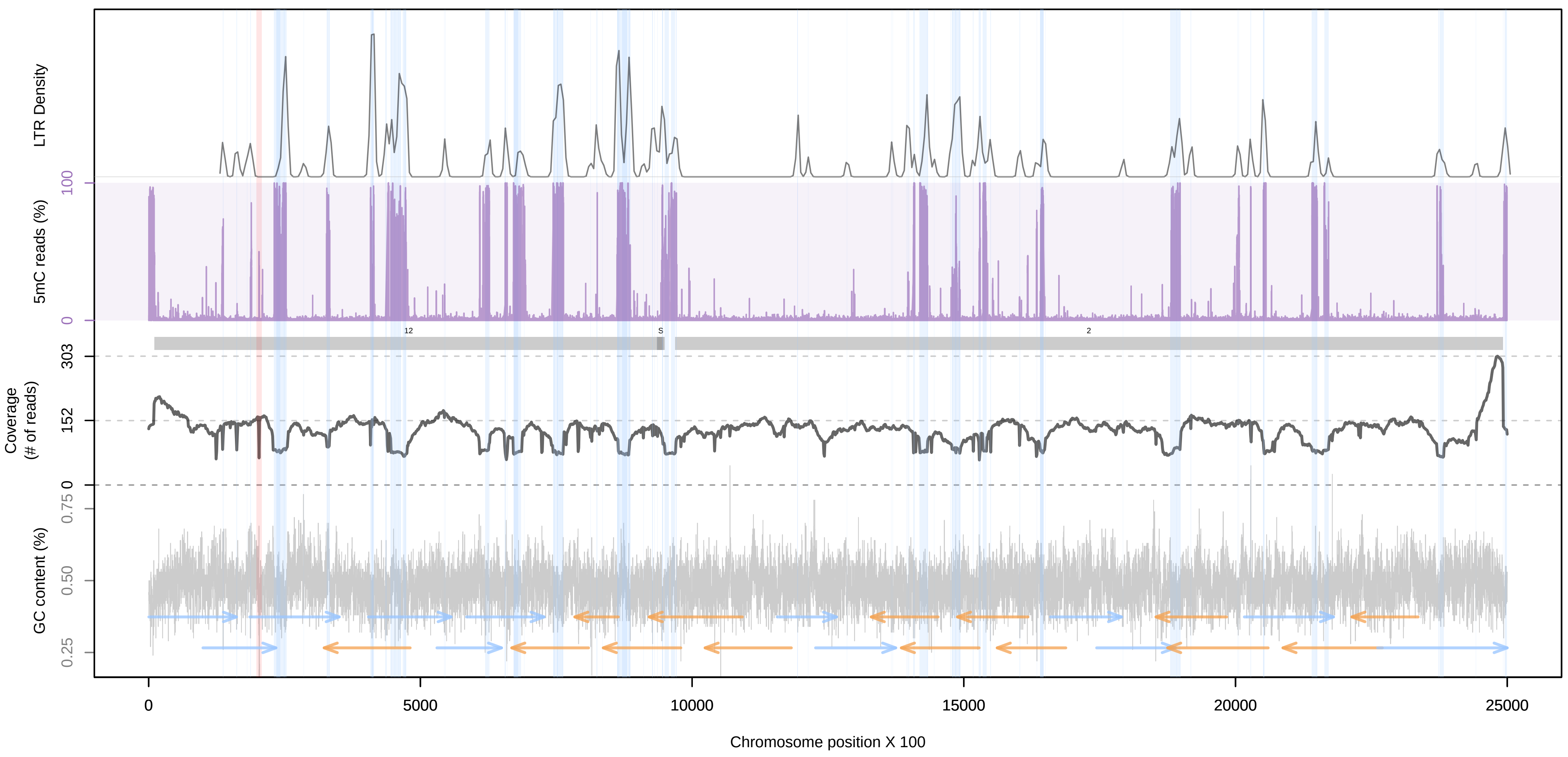

Chromosome 3

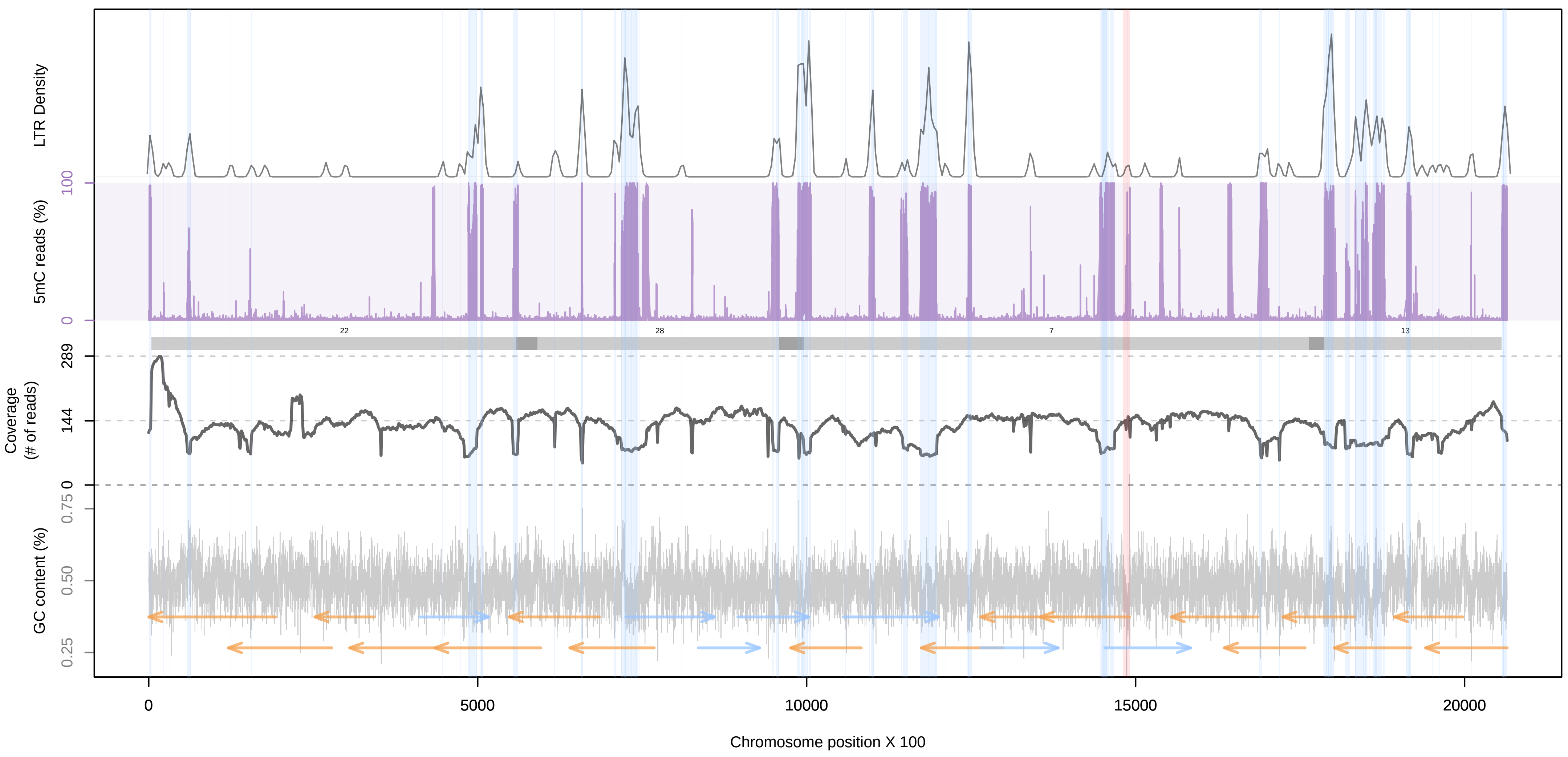

Chromosome 4

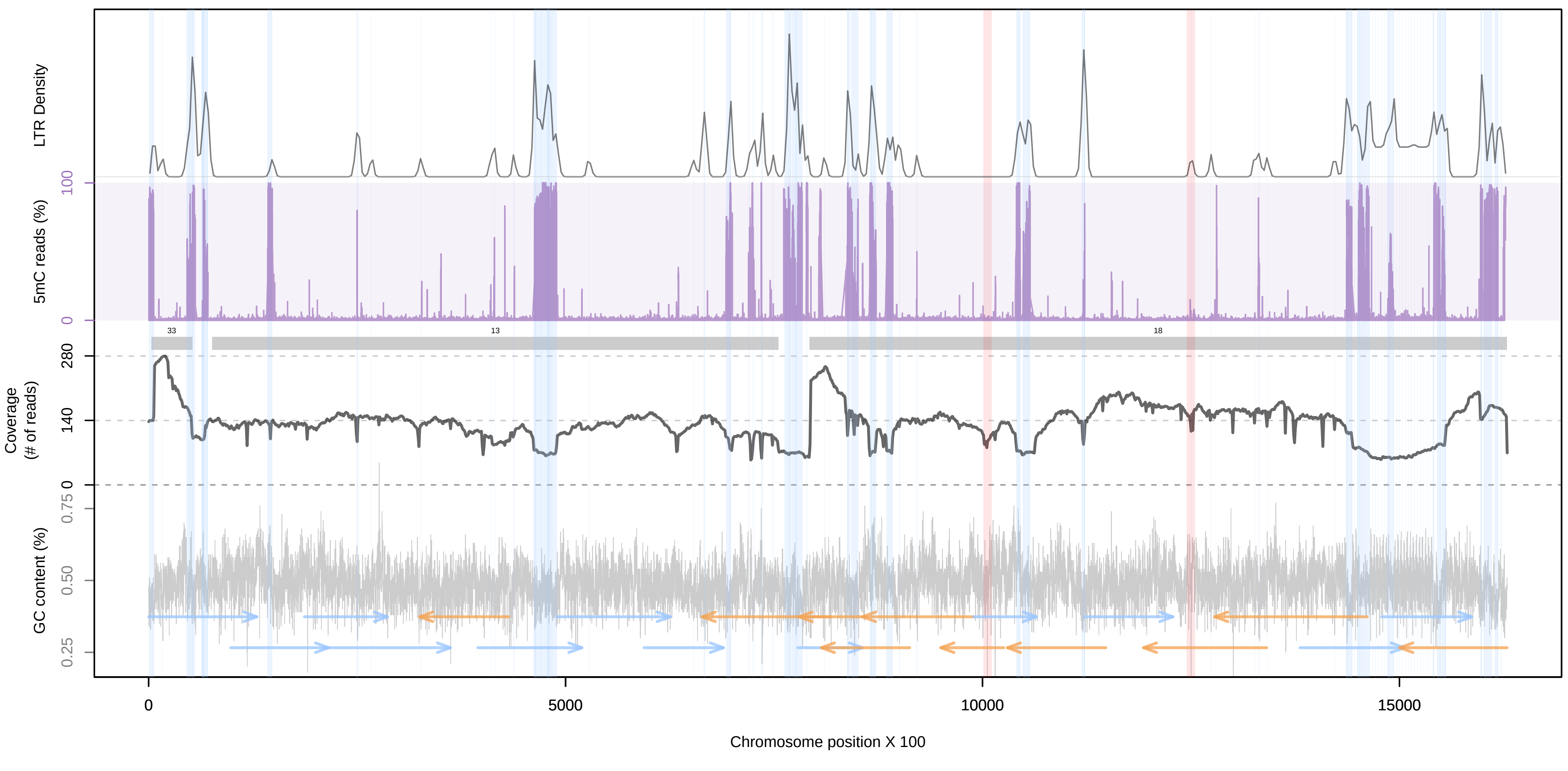

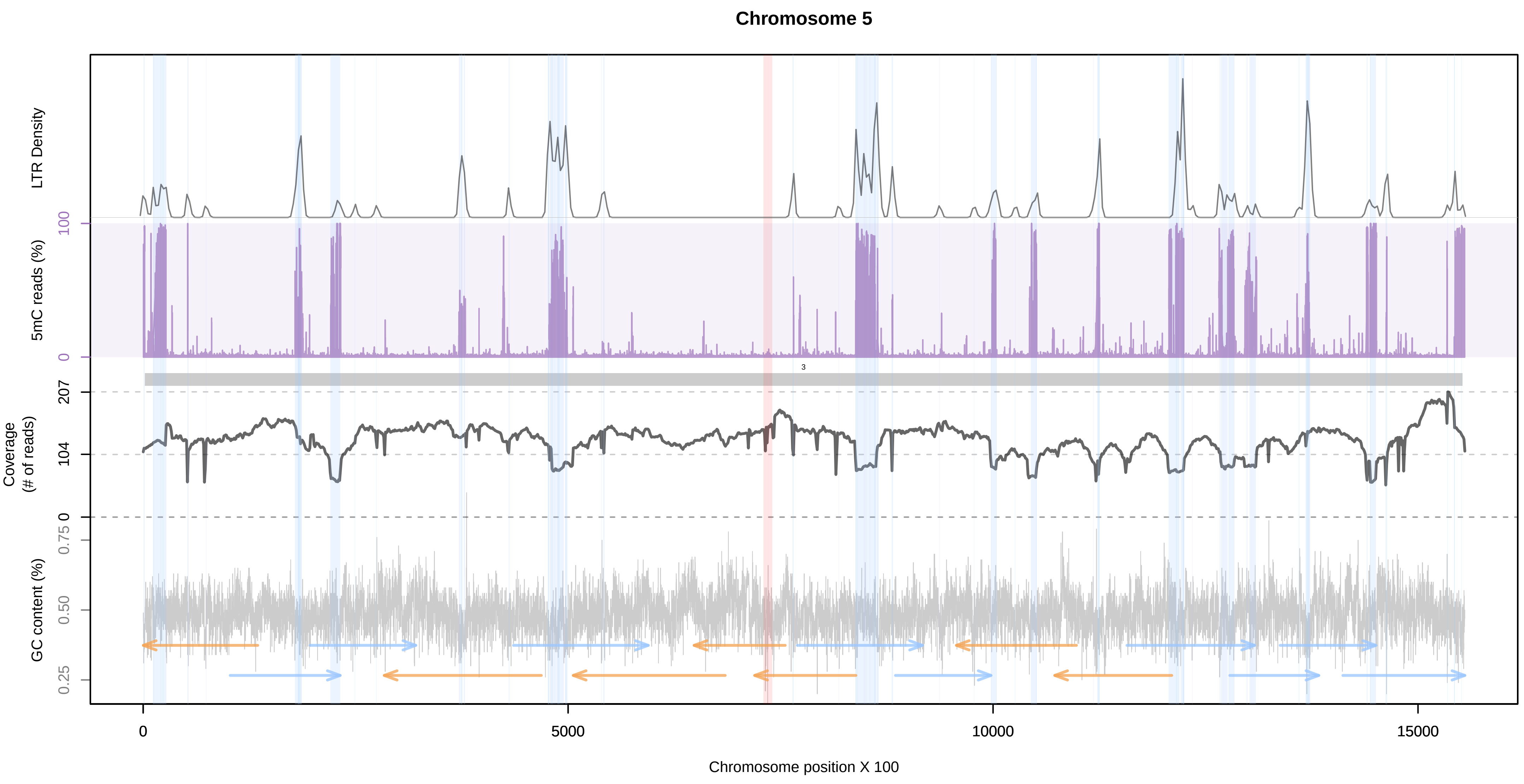
Chromosome 5
LTR Density
5mC reads (%)
100
0
Coverage (# of reads)
207
104
0
GC content (%)
0.75
0.50
0.25
0
5000
10000
15000
Chromosome position X 100

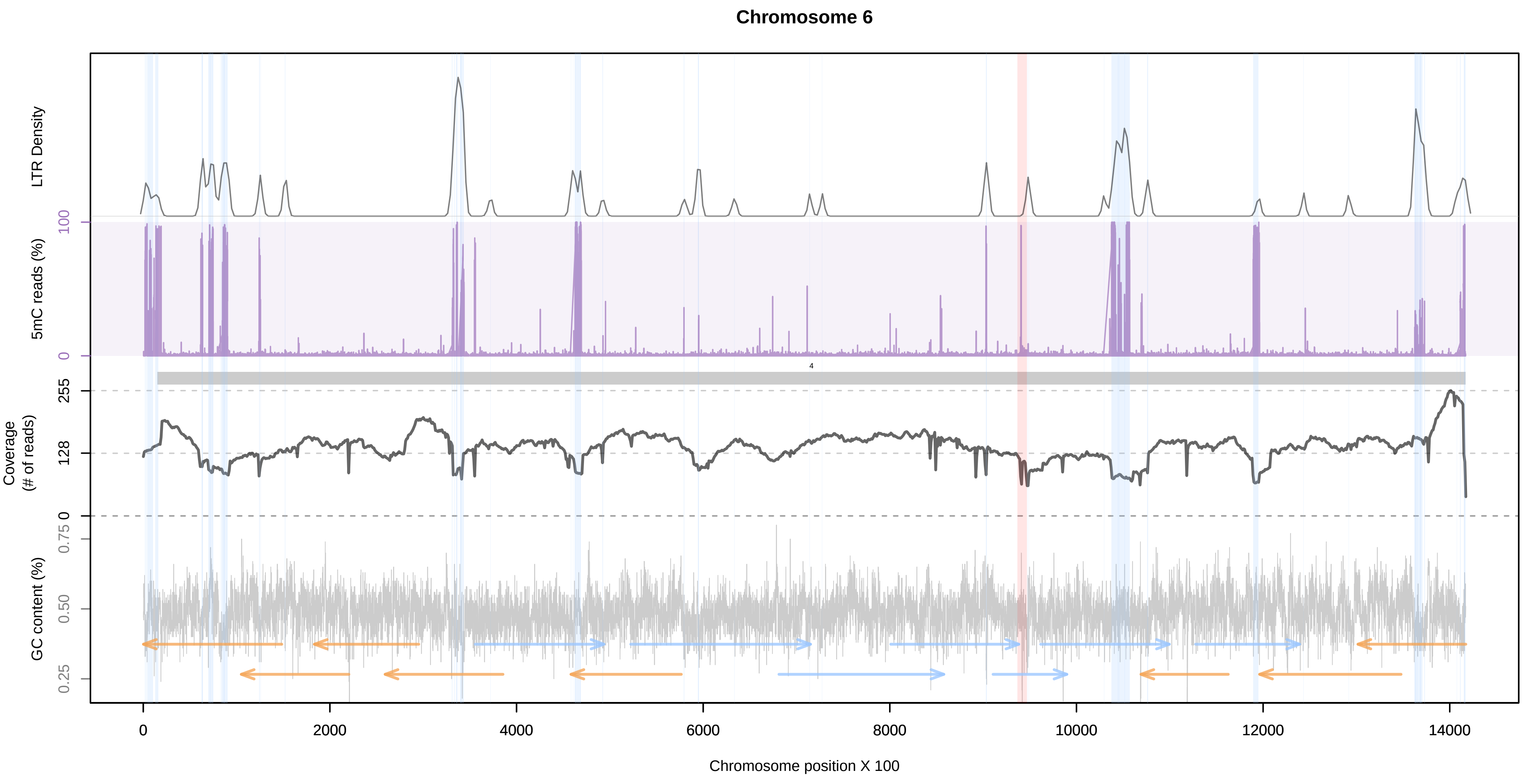
Chromosome 6
LTR Density
5mC reads (%)
100
0
Coverage (# of reads)
255
128
0
GC content (%)
0.75
0.50
0.25
0
2000
4000
6000
8000
10000
12000
14000
Chromosome position X 100

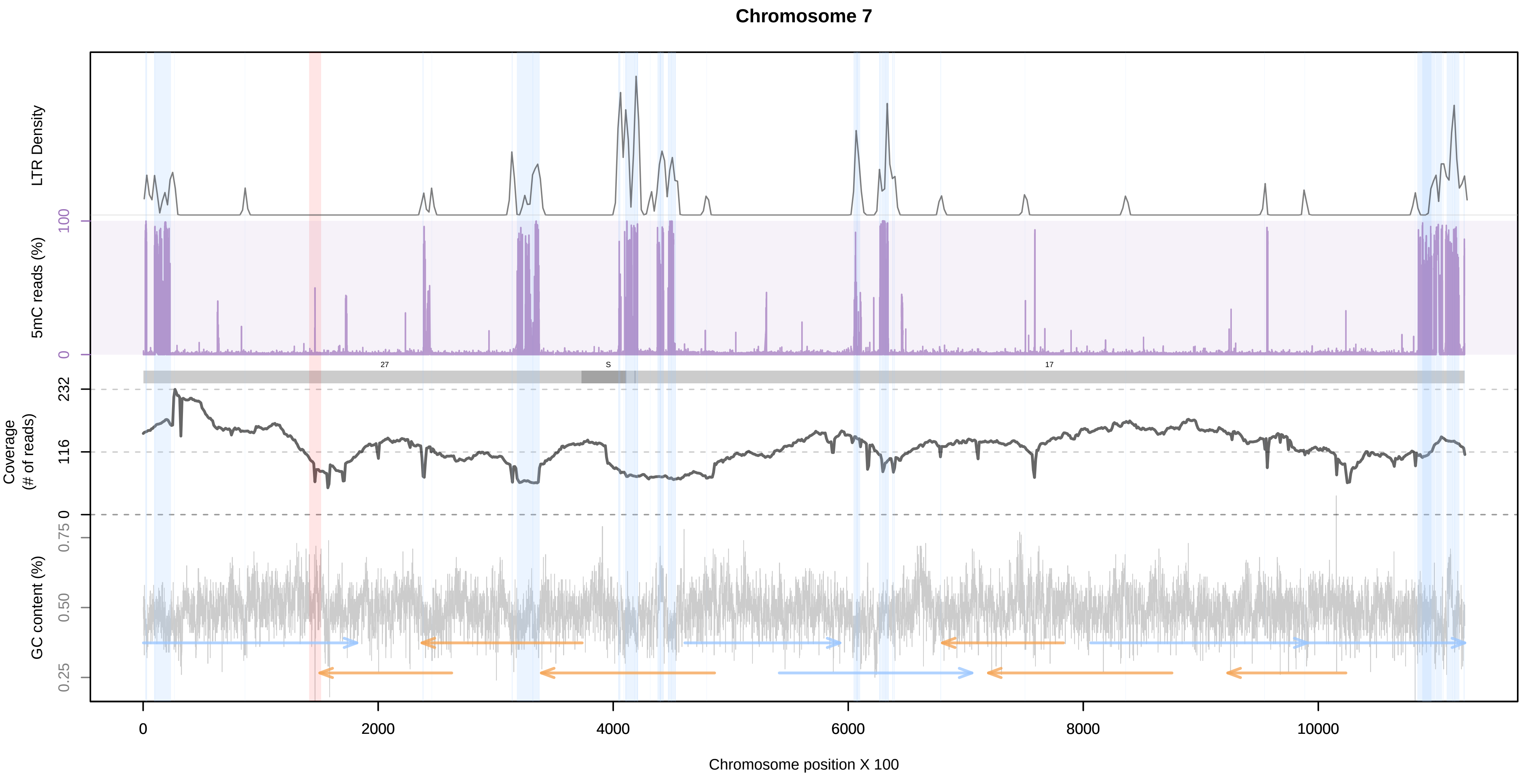
Chromosome 7
LTR Density
5mC reads (%)
100
0
Coverage (# of reads)
232
116
0
GC content (%)
0.75
0.50
0.25
0
2000
4000
6000
8000
10000
Chromosome position X 100

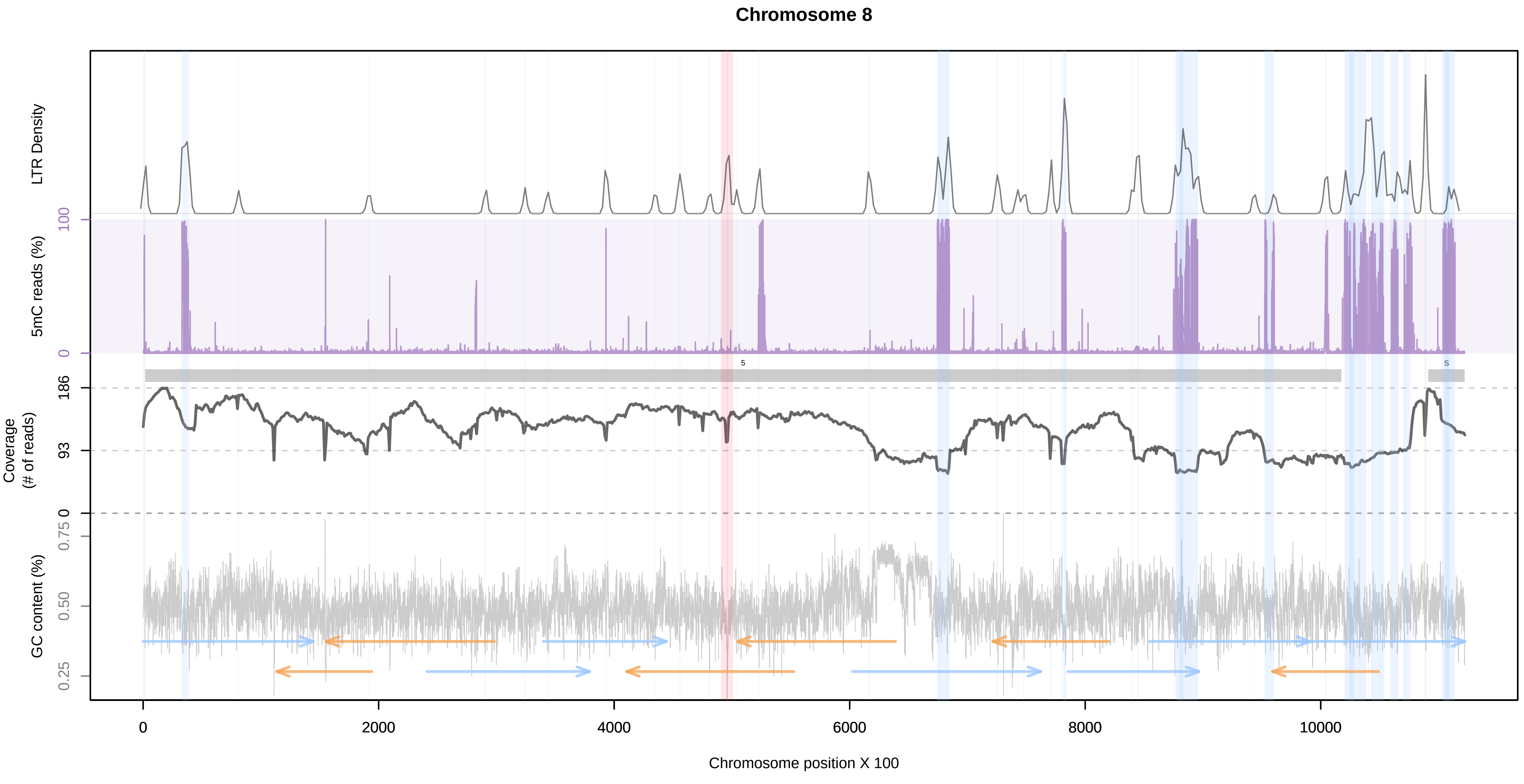
Chromosome 8
LTR Density
5mC reads (%)
100
0
Coverage (# of reads)
186
93
0
GC content (%)
0.75
0.50
0.25
0
2000
4000
6000
8000
10000
Chromosome position X 100

Chromosome 9

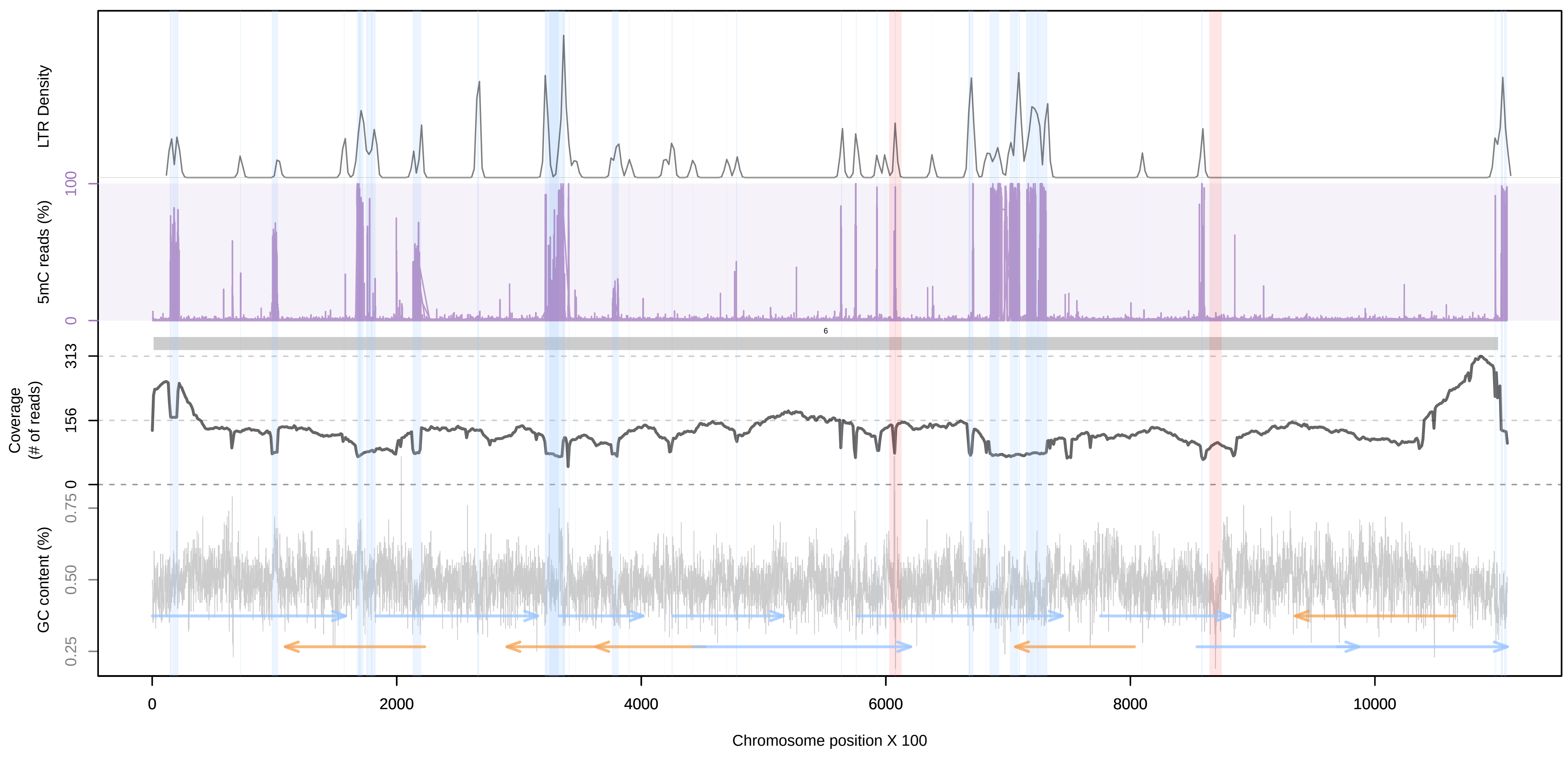

Chromosome 10

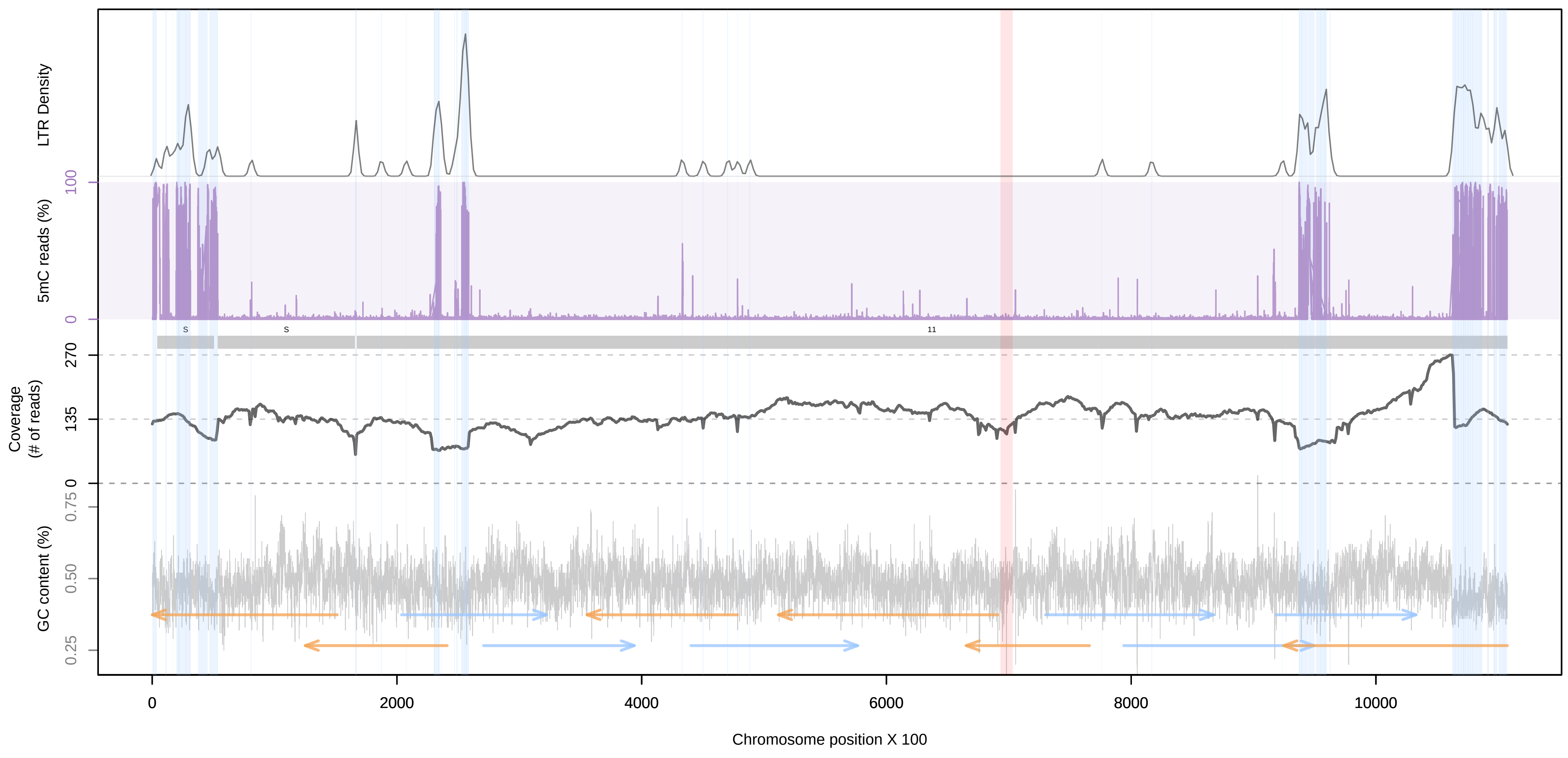

Chromosome 11

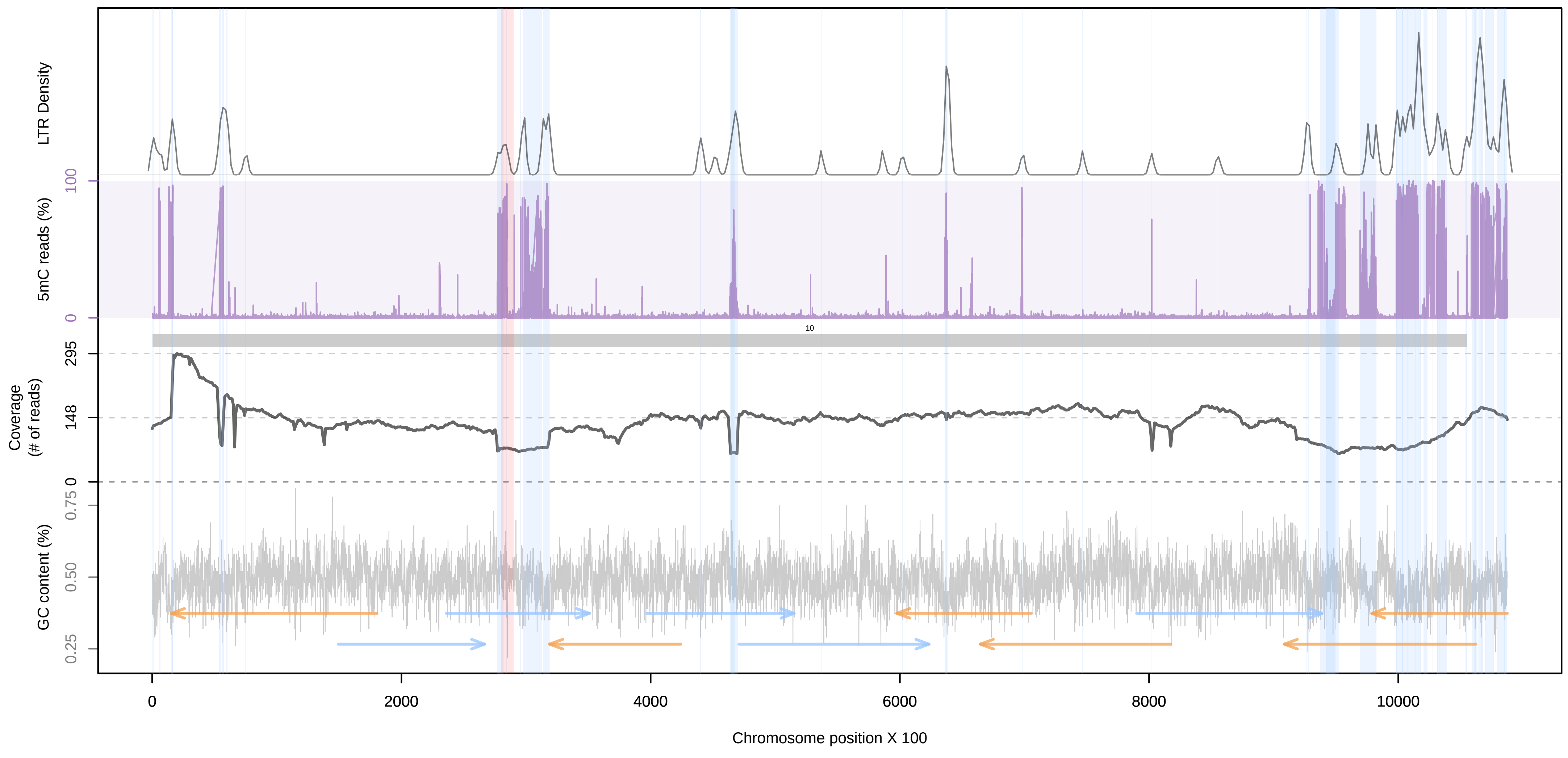

Chromosome 12

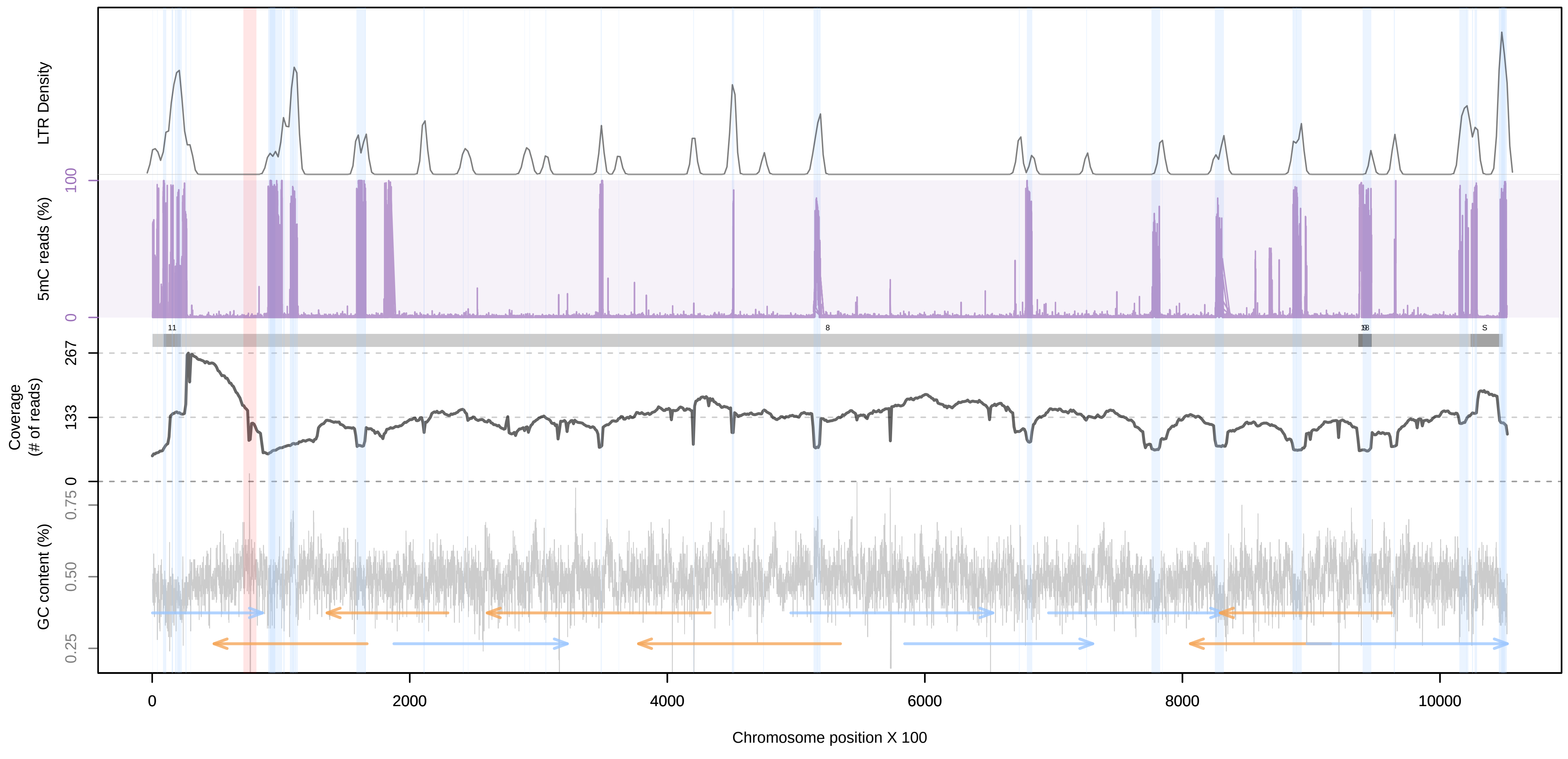

Chromosome 13

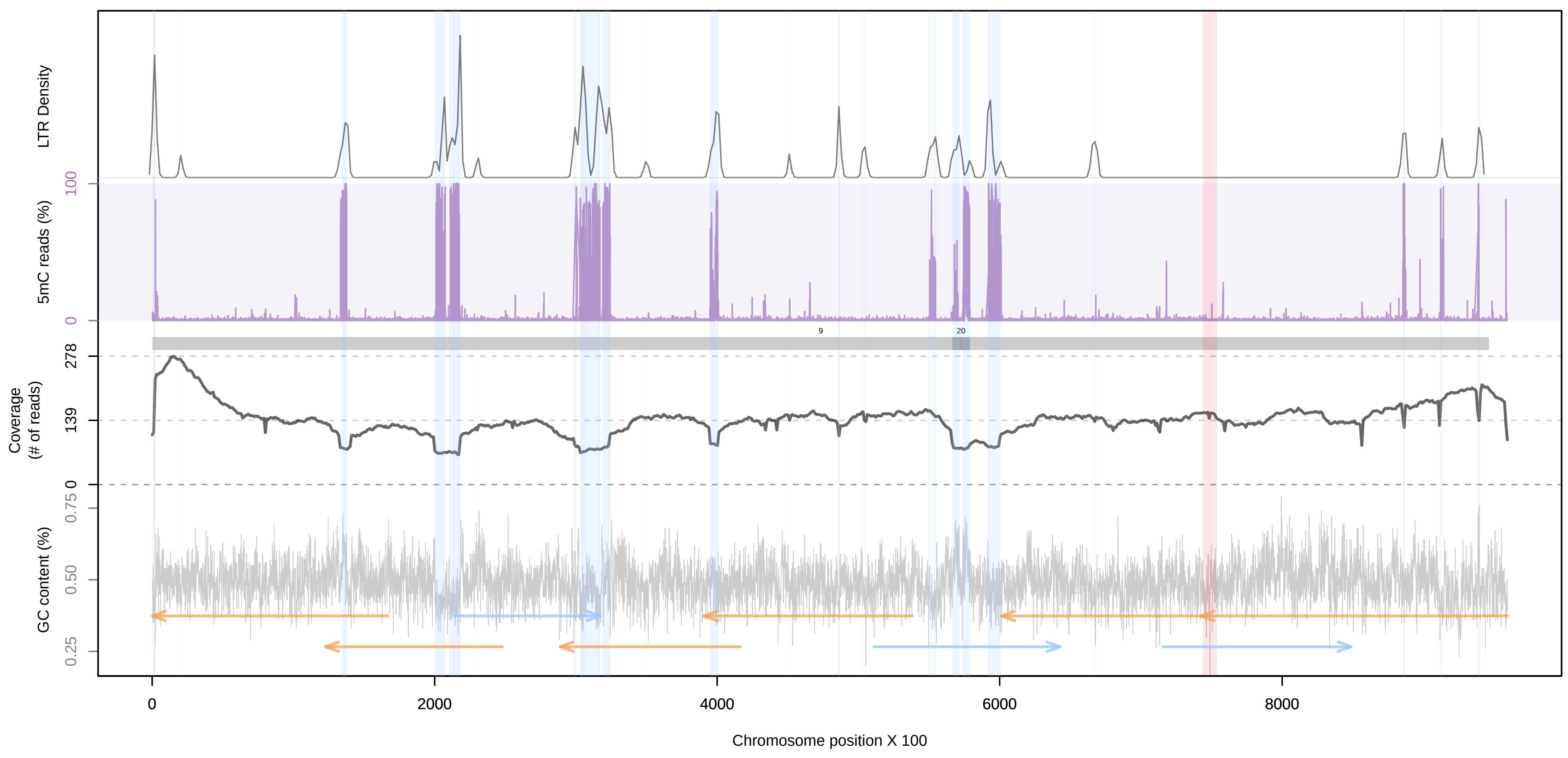

Chromosome 14

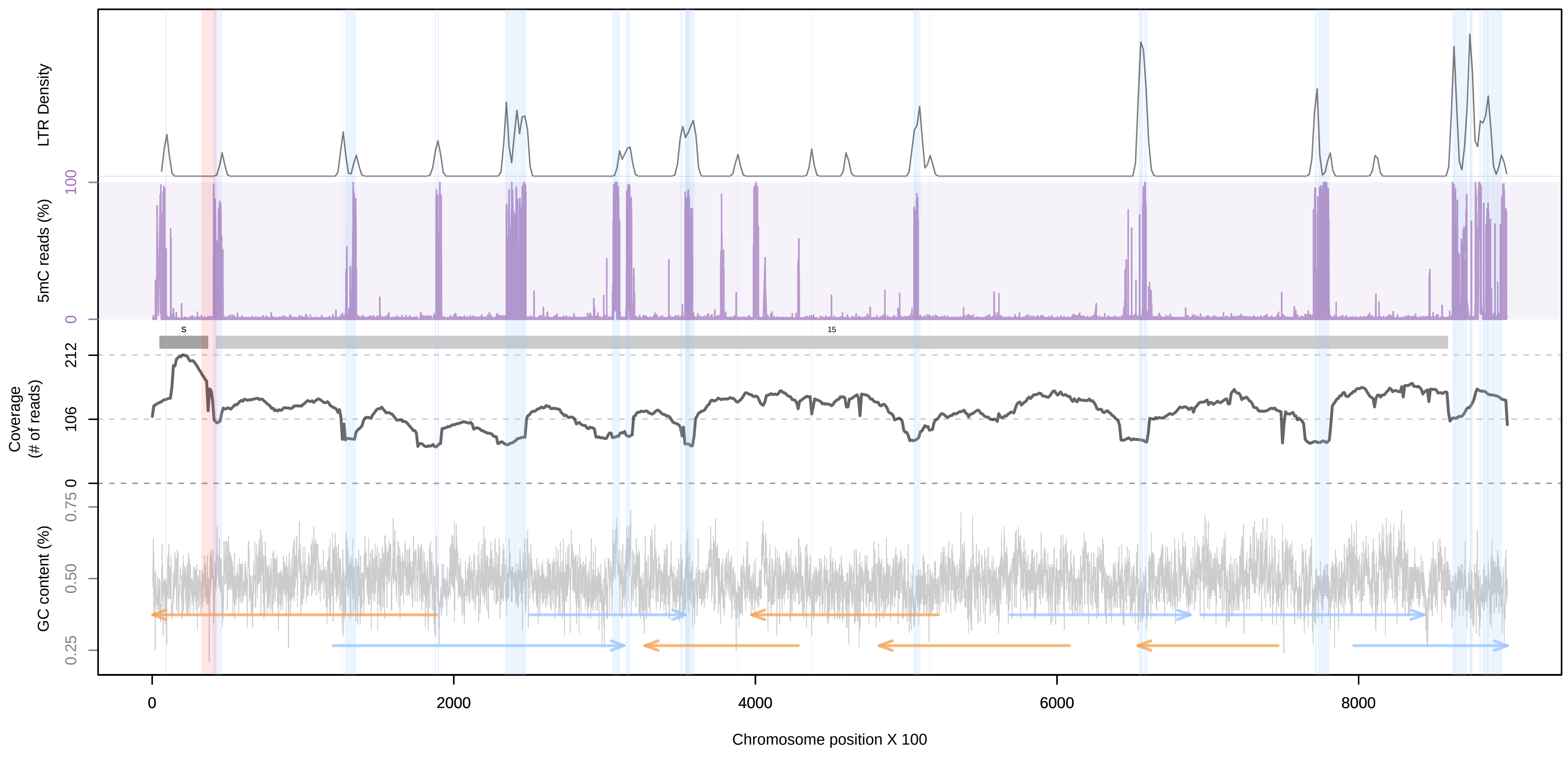

Chromosome 15

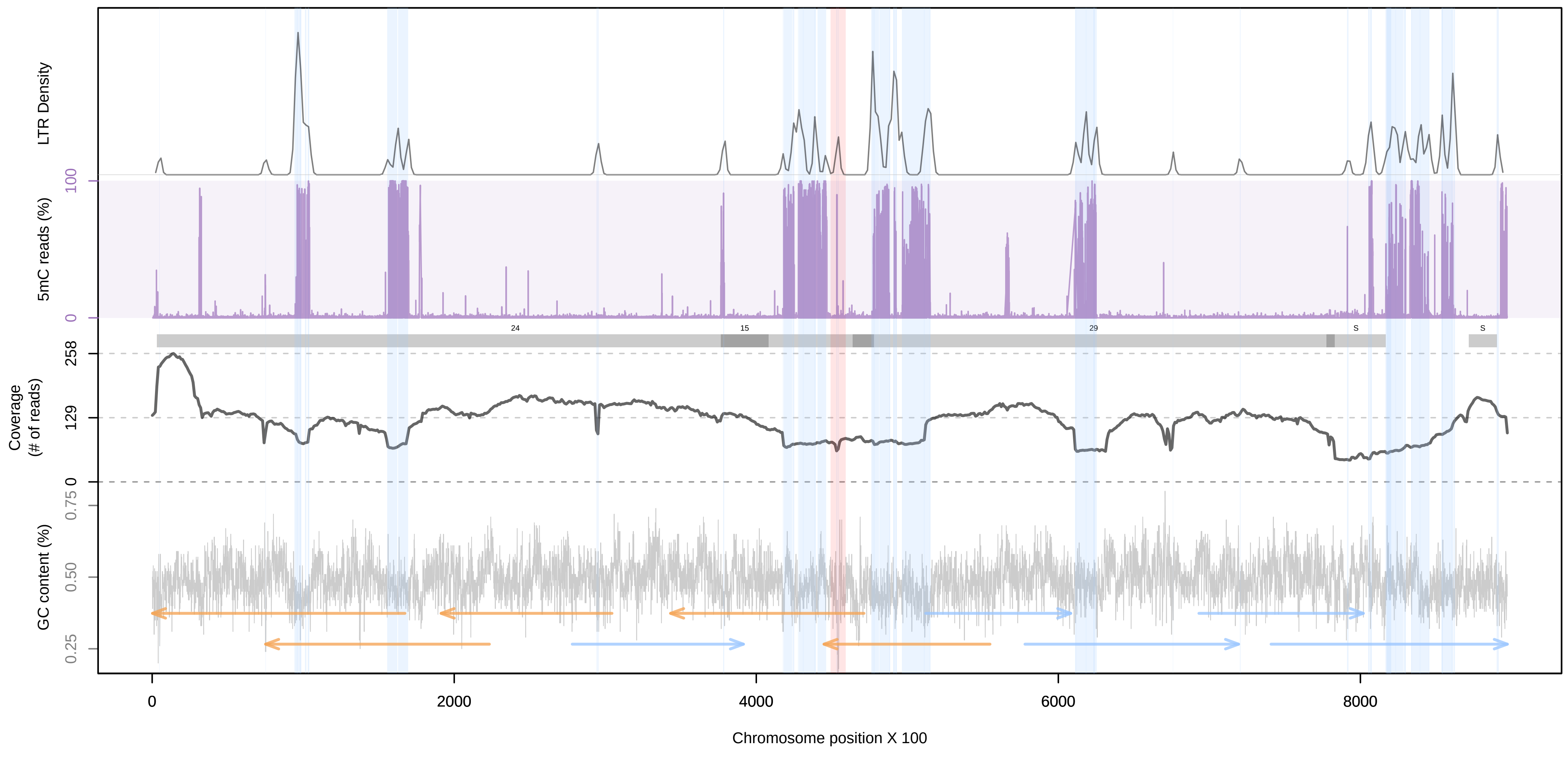

Chromosome 16

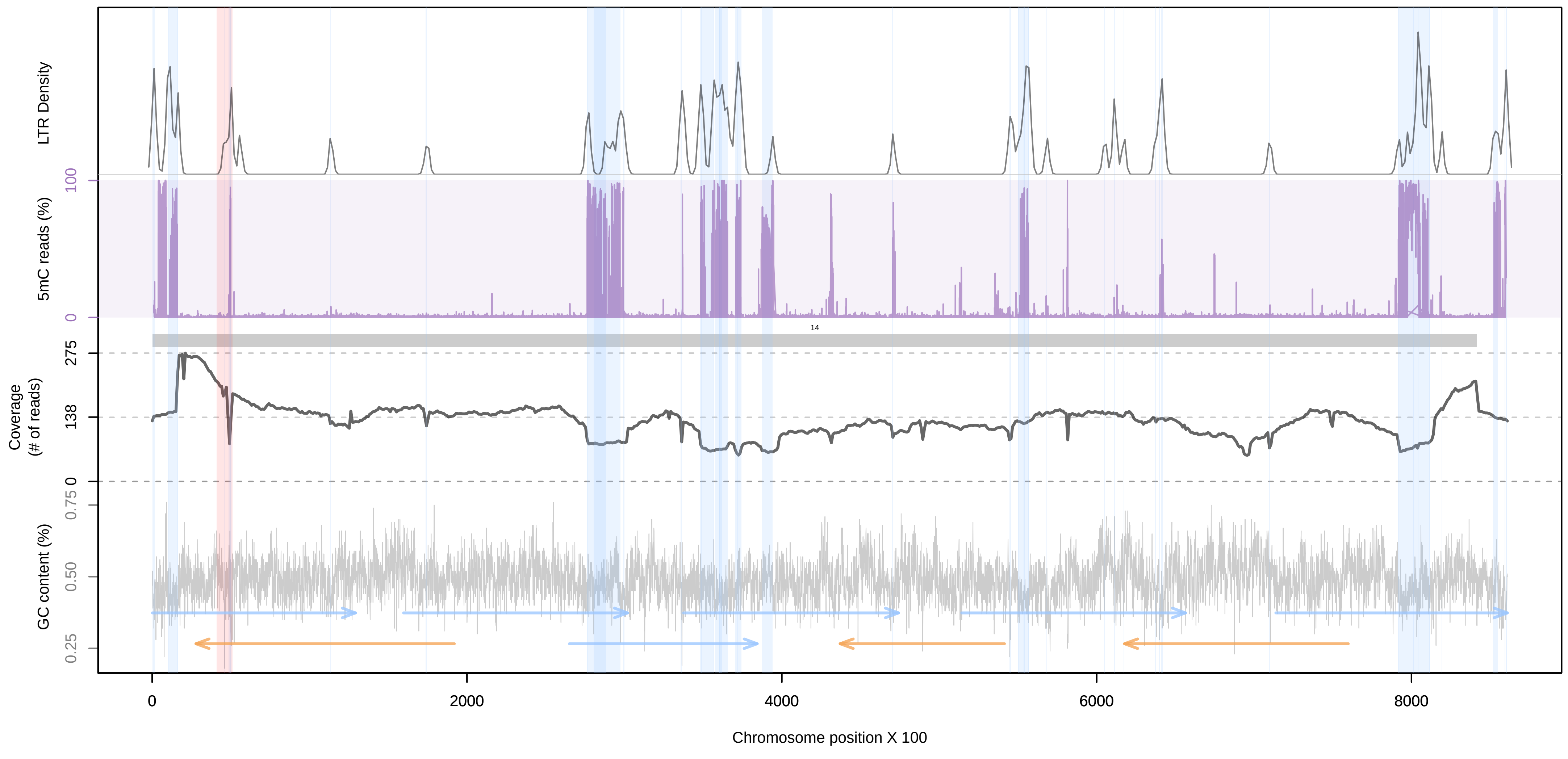

Chromosome 17

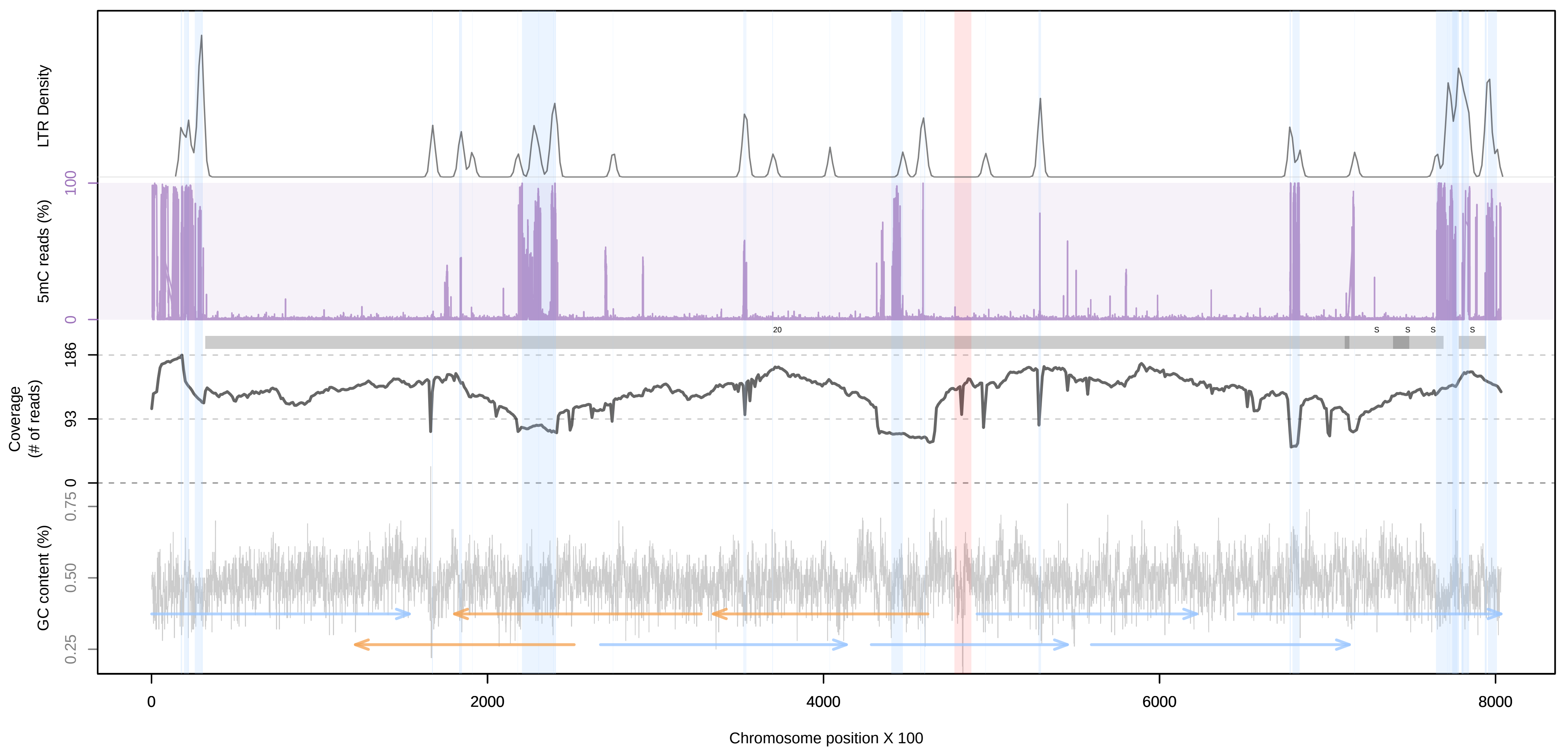

Chromosome 18

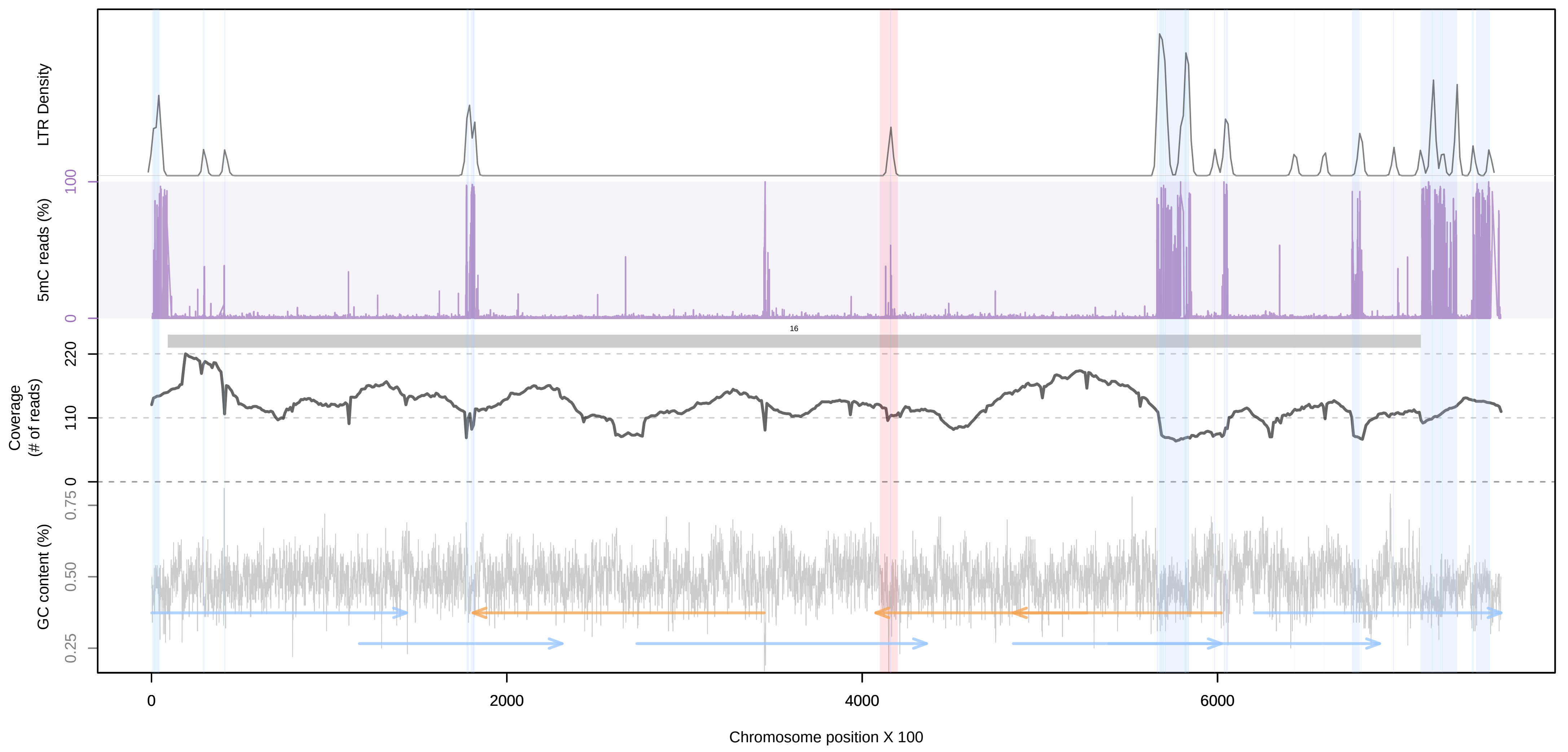

Chromosome 19

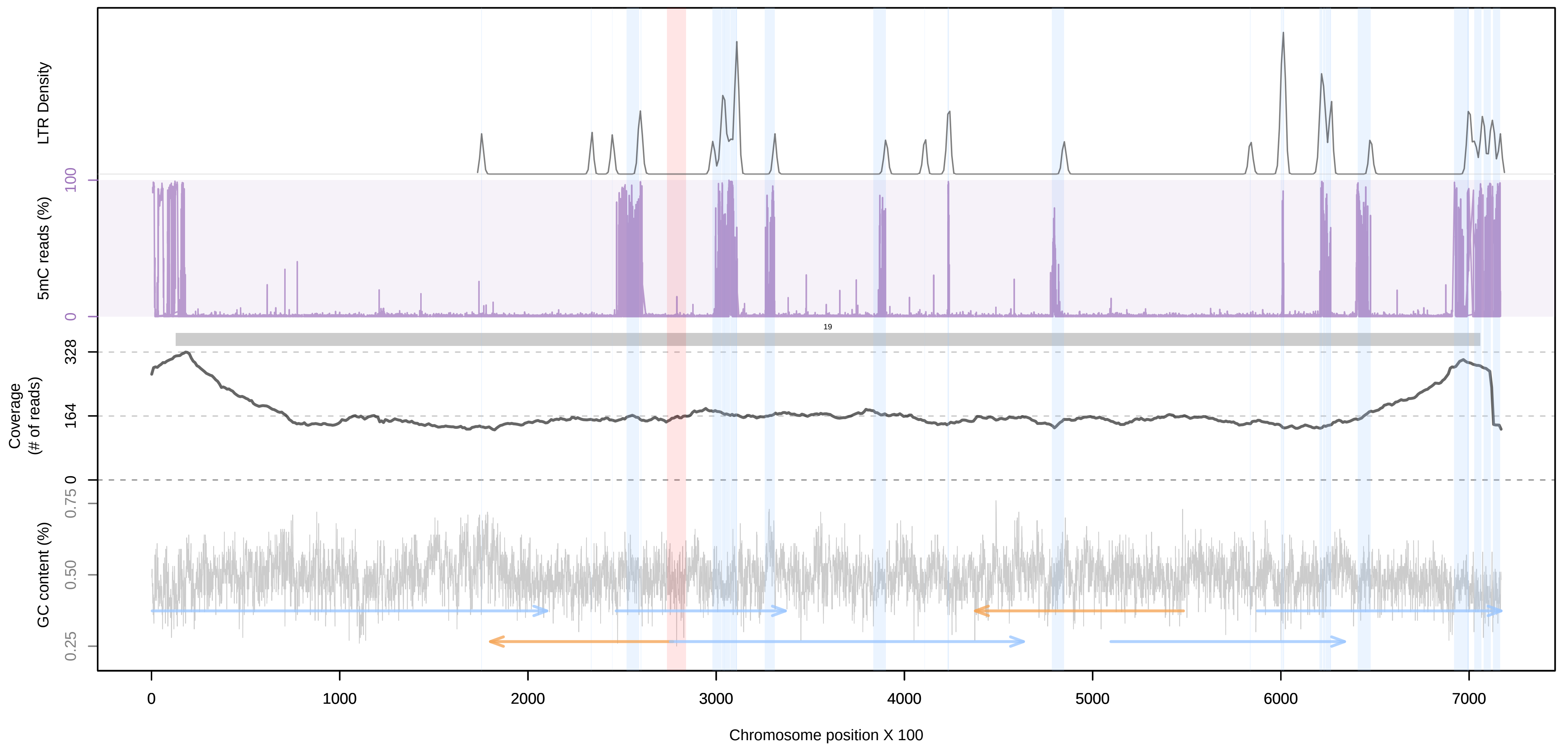

Chromosome 20

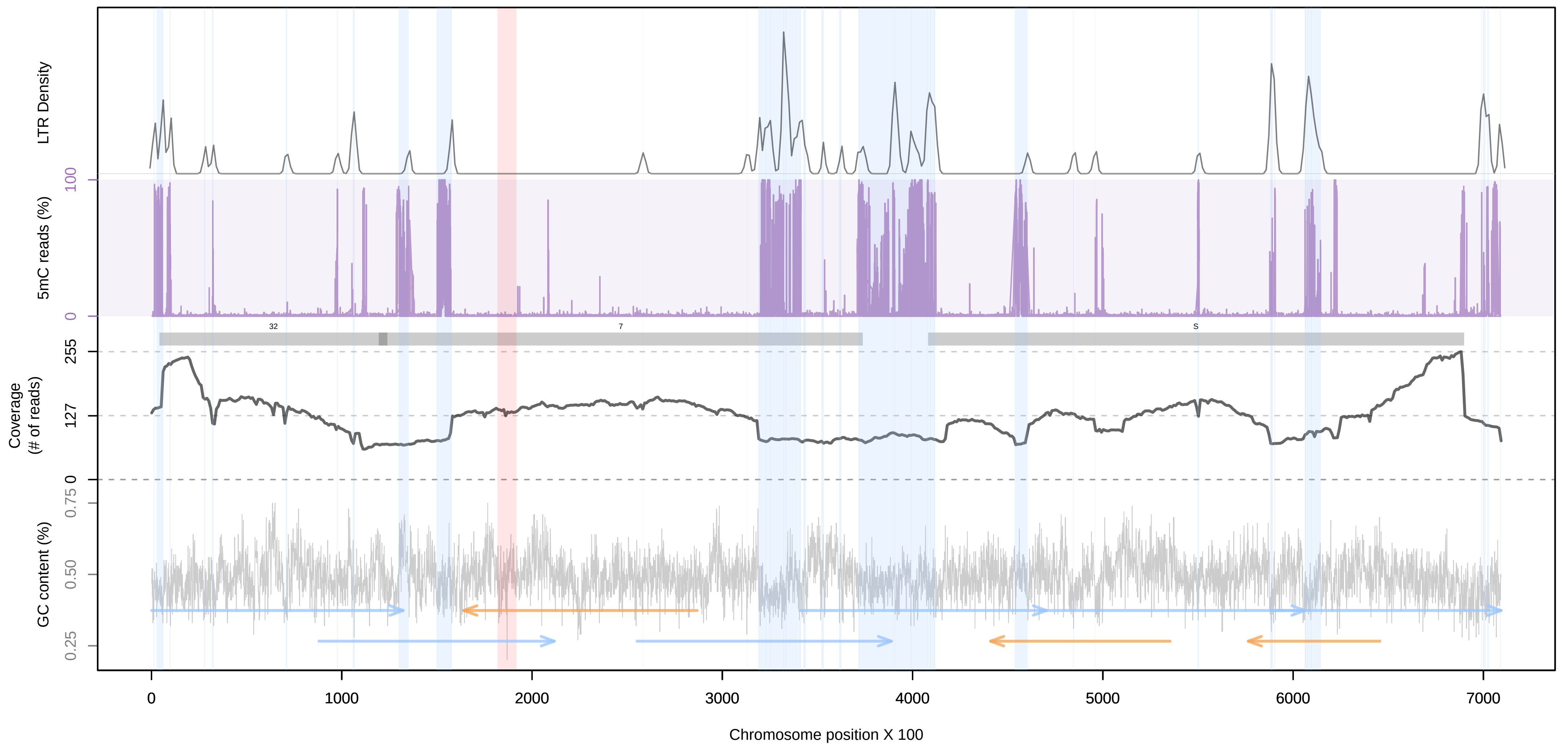

Chromosome 21

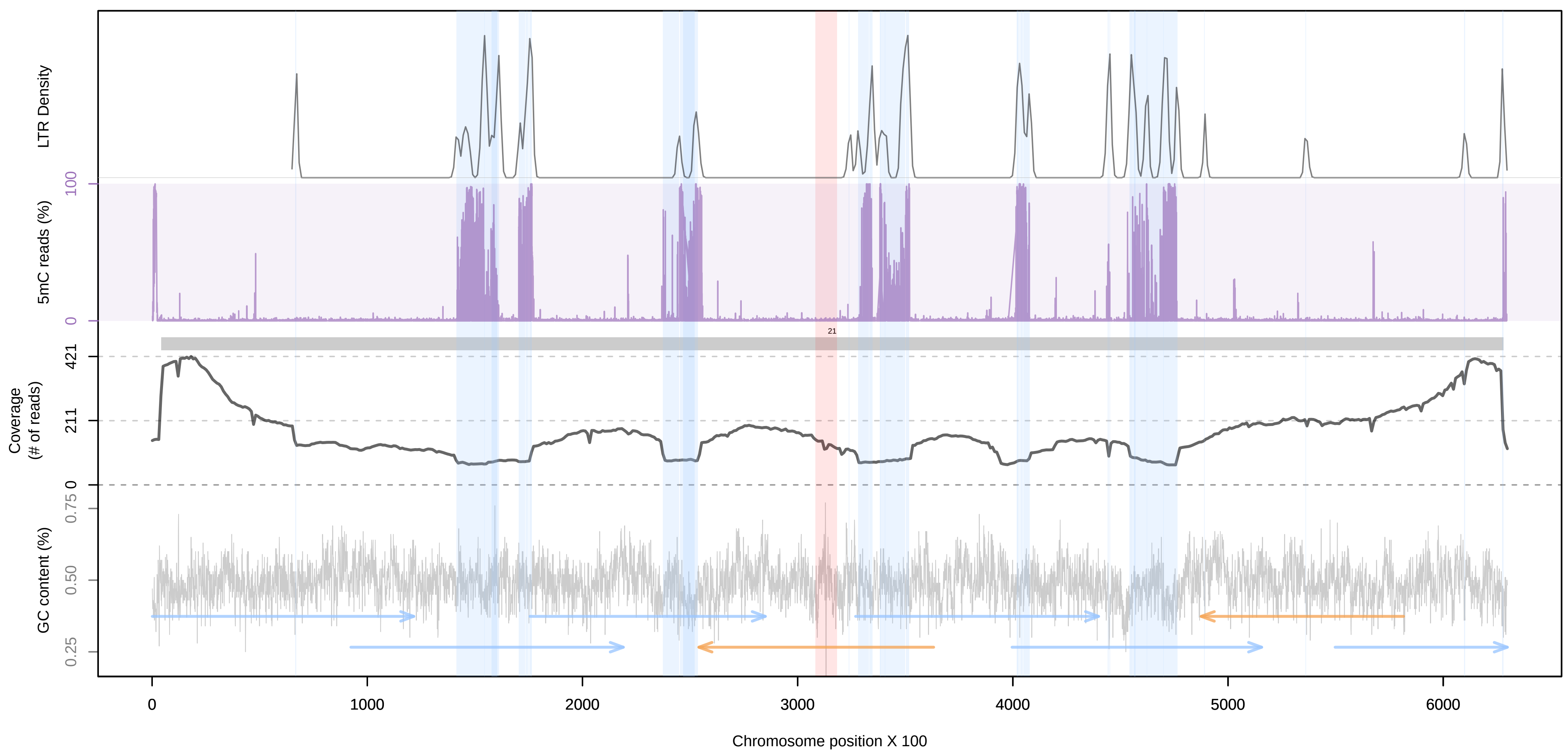

Chromosome 22

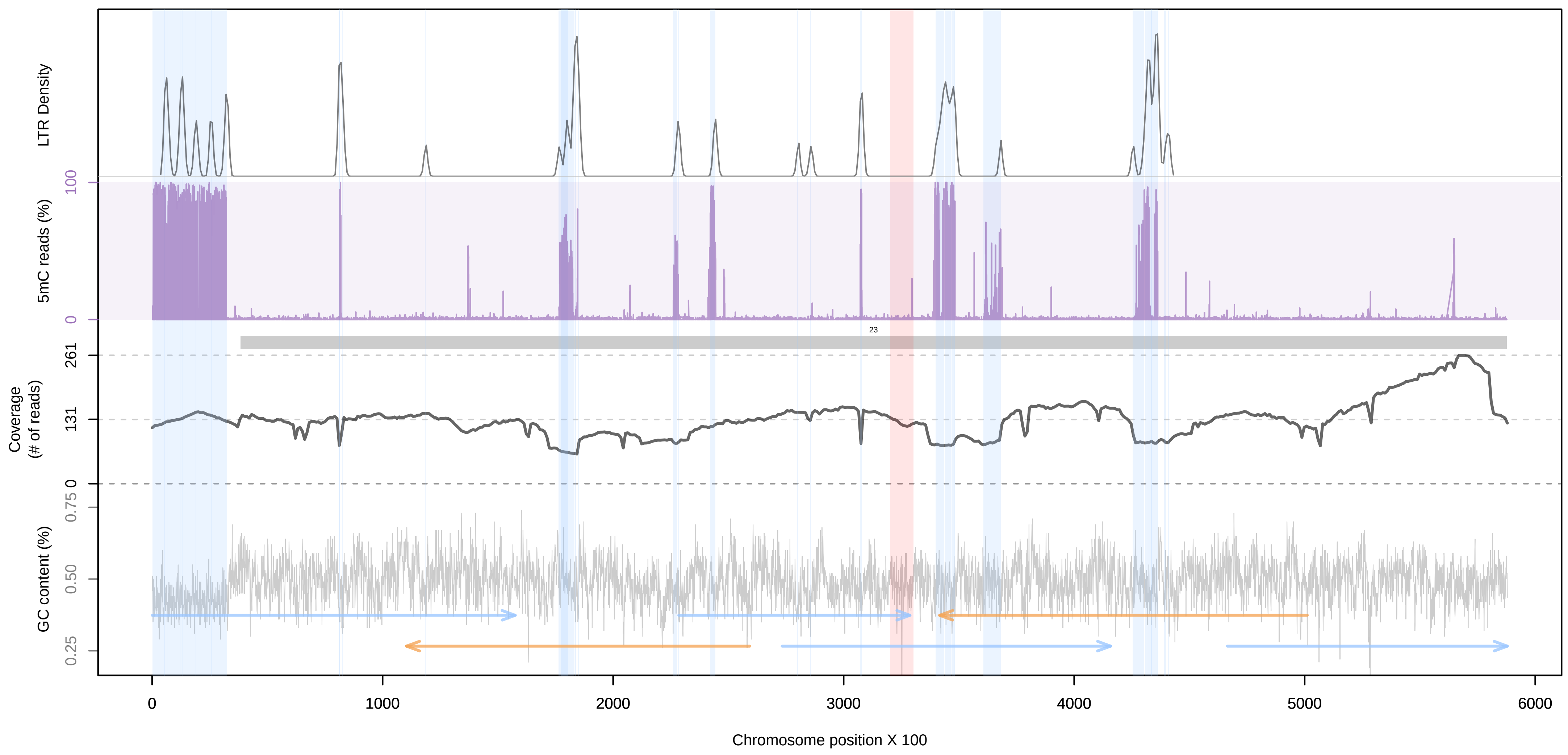

Chromosome 23

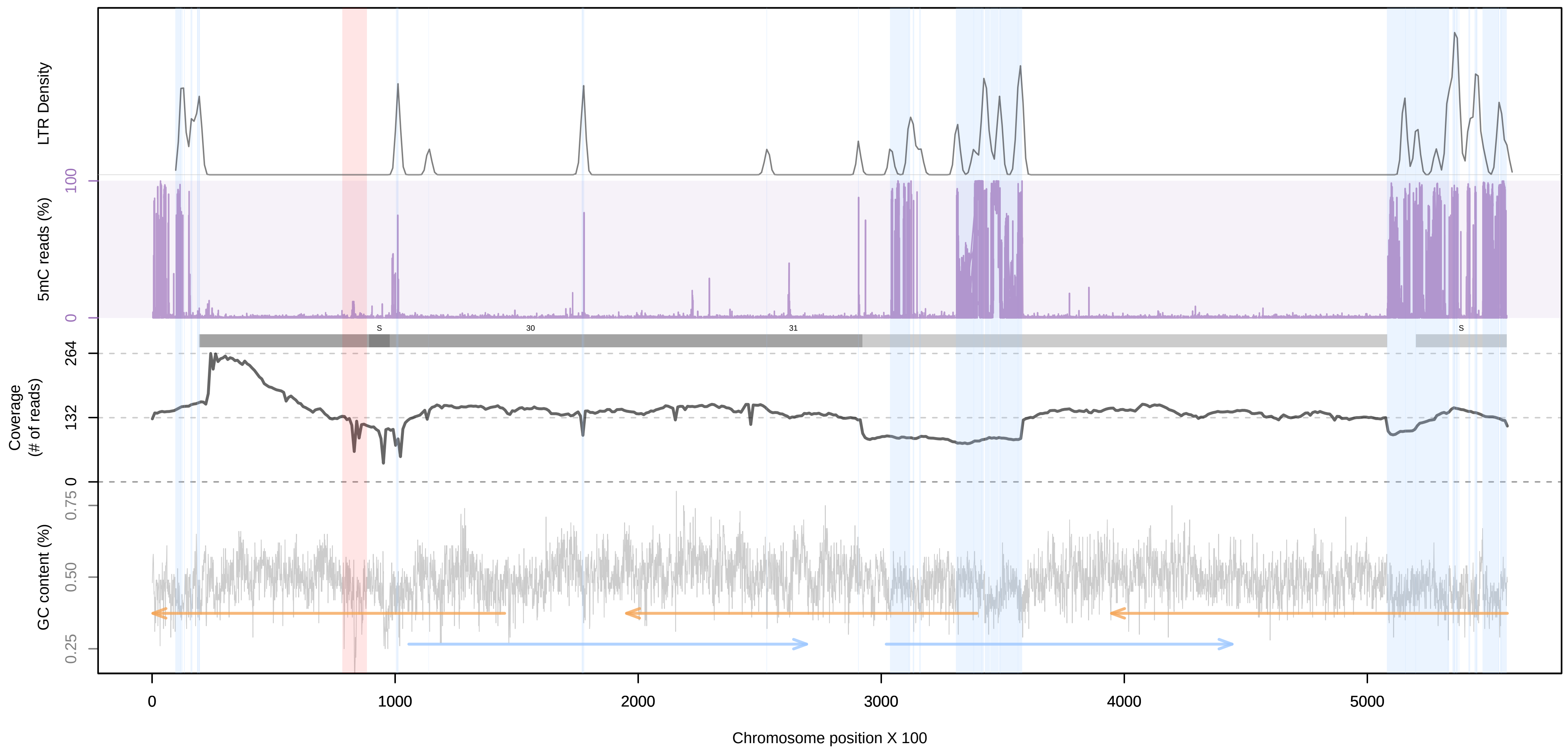

Chromosome 24

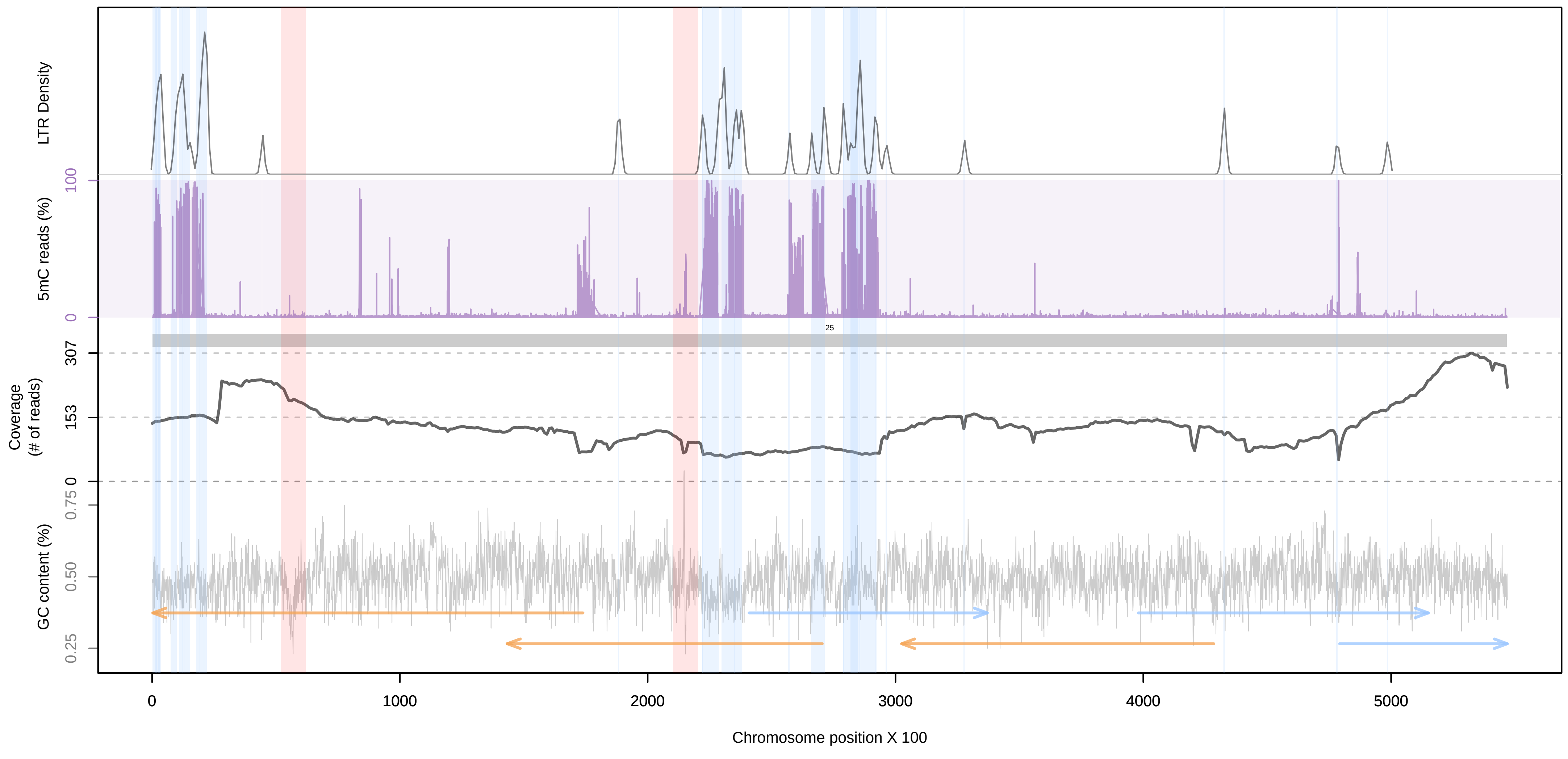

Chromosome 25

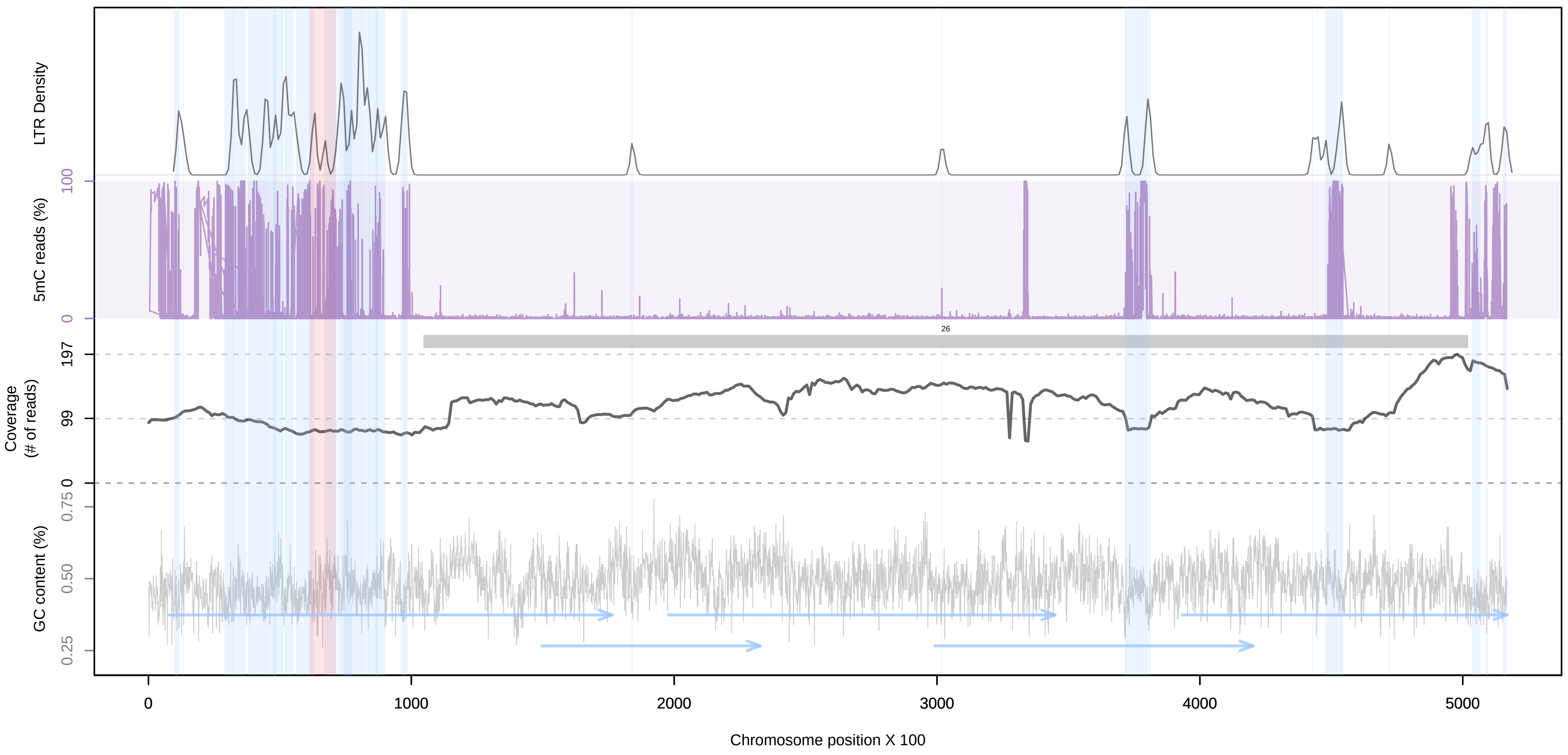

Supplement: Supplemental Information 2 — For each chromosome, the density of LTR-retrotransposons as predicted by the EDTA pipeline are plotted in the top quadrant. The proportion of reads that were called as methylated at each position along the chromosome are plotted in purple. Scaffolds from the previous assembly are overlayed in gray bars, with dark grey representing overlapping regions. Filtered long-read coverage (minimum 20 kb length and 70% query coverage) was plotted as a black line. GC content was calculated and plotted in 100 base windows in the bottom quadrant. An overlapping read tiling path, with a minimum overlap of 30 kb, is shown with orange indicating reads mapping to the negative strand and blue indicating reads mapping to the positive strand. The regions that are annotated at LTR-retrotransposons are highlighted in light blue. [file peerj-10-13607-s002.pdf]
