# Supplementary figures and images for: Telomere-to-telomere genome assembly of Phaeodactylum tricornutum
Source: PeerJ. 2022 Jul 5;10:e13607. doi: 10.7717/peerj.13607 (PMC9266582; doi:10.7717/peerj.13607)

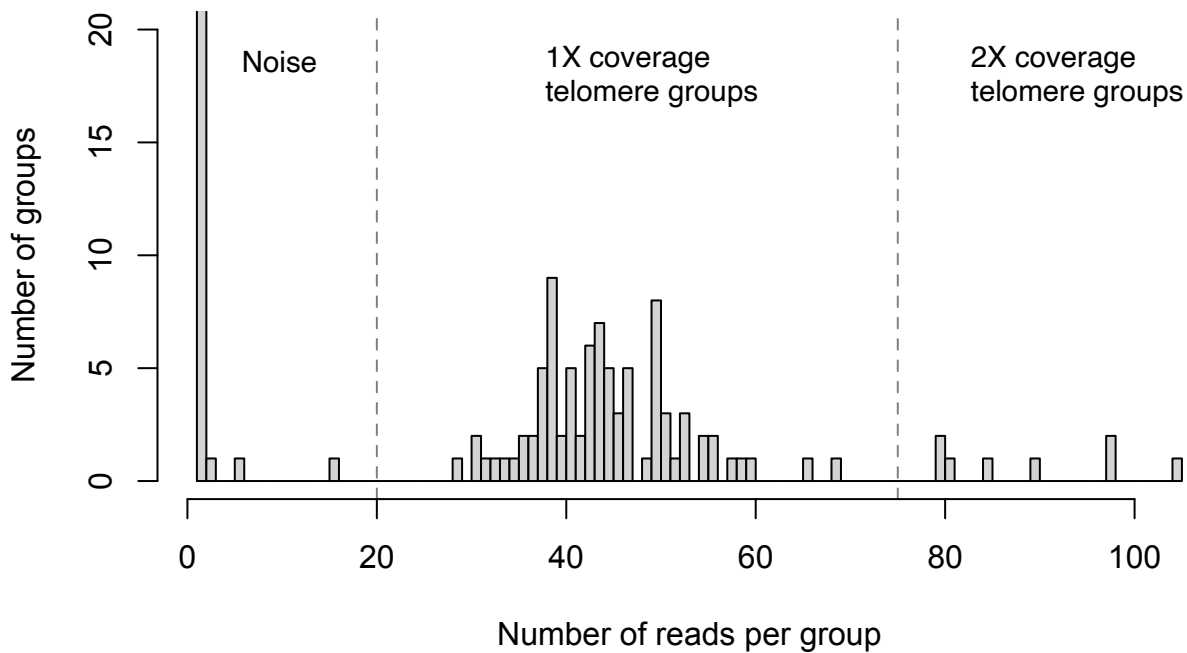

Supplement: Supplemental Information 3 — Telomere graphs were separated into two classes (1) graphs with between 20 and 75 reads, representing normal sequencing depth and (2) graphs with more than 75 reads, representing 2X normal sequencing depth. [file peerj-10-13607-s003.pdf]
